# Supplementary material for: Factors associated with adverse childhood experiences in Scottish children: a prospective cohort study
Source: BMJ Paediatr Open. 2019 Jan 31;3(1):e000340. doi: 10.1136/bmjpo-2018-000340 (PMC6361326; doi:10.1136/bmjpo-2018-000340)
Supplement: Supplementary data [file bmjpo-2018-000340supp001.pdf]

Supplementary Table 1 Source of data used in ACE calculations

**ACE Question**[$]	**Question in GUS**	**Respondent**	**Sweep**						
			1	2	3	4	5	6	7
Sociodemographic<br><br>predictors	Age of mother at birth of first child<br>Equivalised income<br>Education level of mother<br>Household National Statistics Socio-economic Classification<br>Urban/rural classification<br>Scottish Index of Multiple Deprivation (2006)	Parent	X<br><br>X<br>X<br>X<br><br>X<br>X						
1. Did a parent or other adult in the household **often** …<br><br>Swear at you, insult you, put you down, or humiliate you?<br><br>**Or** Act in a way that made you afraid that you might be physically hurt?	*NOT AVAILABLE*	N/A							
2. Did a parent or other adult in the household **often** …<br><br>Push, grab, slap, or throw something at you?<br><br>**Or Ever** hit you so hard that you had marks or were injured?	MGAPQ13 :You slap ^ChildName when ^he has done something wrong – often OR always<br>OR<br>CgPa3 (child int) My parents smack me when I have done something wrong – Often OR always	Parent<br><br><br><br><br>Child							X<br><br><br>X
3. Did an adult or person at least 5 years older than you **ever**…<br><br>Touch or fondle you or have you touch	*ABUSE WAS COVERED UNDER 'ANY OTHER ADVERSE EVENTS' AT TWO SWEEPS AS AN OPEN ENDED QUESTION, HOWEVER FEWER THAN 5 PARENTS LISTED THIS HAPPENING.*	Parent							

their body in a sexual way? **Or** Try to or actually have oral, anal, or vaginal sex with you?									
4. Did you **often** feel that …<br><br>No one in your family loved you or thought you were important or special?<br><br>**Or** Your family didn't look out for each other, feel close to each other, or support each other?	(child interview)<br>CgPa2: My parents ask about my day in school – NEVER<br>OR<br>CgPa4: My parents play games or do other fun things with me – NEVER<br>OR<br>CgPa6: My parents help me with my homework – NEVER<br>OR<br>CgPa7: My parents tell me when I'm doing a good job with something- NEVER<br>OR<br>CgPa13: My parents check to make sure I'm doing ok - NEVER	Child							X
5. Did you **often** feel that …<br><br>You didn't have enough to eat, had to wear dirty clothes, and had no one to protect you?<br><br>**Or** Your parents were too drunk or high to take care of you or take you to the doctor if you needed it?	*NOT AVAILABLE*	N/A							
6. Were your parents **ever** separated or divorced?	If only one natural parent in the household at any sweep (MxHGnp01)	Parent	X	X	X	X	X	X	X
7. Was your mother or stepmother:	IF respondent is mother/stepmother AND IF:<br>And since ^childname was born, has any partner or ex-	Parent						X	

**Often** pushed, grabbed, slapped, or had something thrown at her?<br><br>**Or Sometimes or often** kicked, bitten, hit with a fist, or hit with something hard?<br><br>**Or Ever** repeatedly hit over at least a few minutes or threatened with a gun or knife?	partner ever done any of these things to you?<br>*MfHdv061* Pushed you or held you down – YES<br>OR<br>*MfHdv062* Kicked, bitten or hit you – YES<br>OR<br>*MfHdv064* Used a weapon against you, for example an ashtray or a bottle – YES<br><br>AND<br><br>*MfHdv07* How many times since ^childname was born have any of these things happened to you? - 3. Four or five times OR 4. Six or more times OR 5. Too many to count<br><br>OR<br><br>*MfHdv034* Threatened you with a weapon, for example an ashtray or a bottle - YES								
8. Did you live with anyone who was a problem drinker or alcoholic or who used street drugs?	Has ^childname experienced any of the things listed on this card since the last interview?<br>*MgOve15* Drug taking/alcoholism in the immediate family - YES<br>Alcohol level >14 units per week (harmful level at time)<br>Use of drugs (use any street drugs in prev 12 months)	Parent	X<br>X	X	X<br>X		X<br>X	X	X
9. Was a household member depressed or mentally ill or did a household member attempt suicide?	Has ^childname experienced any of the things listed on this card since the last interview?<br>*MgOve16* Mental disorder in the immediate family – YES<br>OR<br>DASS* score of >1SD above 0<br>OR<br>SF12MCS** score of <=35	Parent	X	X	X	X	X	X	X
10. Did a household member go to prison?	Has ^childname experienced any of the things listed on this card since the last interview?	Parent		X			X	X	X

	MgOve20 Parent in prison - YES<br>OR (where available)<br>*MxHGaww3/4*								

*DASS: Depression, Anxiety and Stress Scale
**SF12MCS: Short Form 12 Mental Component Scale
[$]These are the same 10 ACEs used in the comprehensive Wave 2 of the original ACE Study (Dong et al., 2004).
