# Supplementary material for: Factors associated with adverse childhood experiences in Scottish children: a prospective cohort study
Source: BMJ Paediatr Open. 2019 Jan 31;3(1):e000340. doi: 10.1136/bmjpo-2018-000340 (PMC6361326; doi:10.1136/bmjpo-2018-000340)
Supplement: Supplementary data [file bmjpo-2018-000340supp002.pdf]

Supplementary Table 2 Spearman correlations between socio-demographic data

	1.	2.	3.	4.	5.	6.
1. Sex	1	0.03*	0.01	-0.01	-0.01	<0.01
2. Age of mother at birth of child		1	0.50**	-0.42**	-0.36**	0.04
3. Household income quintiles			1	-0.51**	-0.49**	0.04*
4. Respondent education				1	0.38**	-0.06**
5. Scottish Index of Multiple Deprivation Quintiles					1	-0.18**
6. Urban-rural classification						1

*correlation is significant at the 0.05 level

** correlation is significant at the 0.01 level

r_s	*Interpretation*
0.00-0.19	Very weak
0.20-0.39	Weak
0.40-0.59	Moderate
0.60-0.79	Strong
0.80-1.0	Very strong
