# Supplementary material for: Factors associated with adverse childhood experiences in Scottish children: a prospective cohort study
Source: BMJ Paediatr Open. 2019 Jan 31;3(1):e000340. doi: 10.1136/bmjpo-2018-000340 (PMC6361326; doi:10.1136/bmjpo-2018-000340)
Supplement: Author's manuscript [file bmjpo-2018-000340.draft_revisions.pdf]

BMJ Paediatrics Open

BMJ Paediatrics Open is committed to open peer review. As part of this commitment we make the peer review history of every article we publish publicly available.

When an article is published we post the peer reviewers' comments and the authors' responses online. We also post the versions of the paper that were used during peer review. These are the versions that the peer review comments apply to.

The versions of the paper that follow are the versions that were submitted during the peer review process. They are not the versions of record or the final published versions. They should not be cited or distributed as the published version of this manuscript.

BMJ Paediatrics Open is an open access journal and the full, final, typeset and author-corrected version of record of the manuscript is available on our site with no access controls, subscription charges or pay-per-view fees (http://bmjpaedsopen.bmj.com).

If you have any questions on BMJ Paediatrics Open's open peer review process please email info.bmjpo@bmj.com

BMJ Paediatrics Open

Confidential: For Review Only

What factors are associated with Adverse Childhood Experiences in Scottish children: results from a birth cohort study

Journal:	*BMJ Paediatrics Open*
Manuscript ID	bmjpo-2018-000340
Article Type:	Original article
Date Submitted by the Author:	03-Jul-2018
Complete List of Authors:	Marryat, Louise; University of Edinburgh, Farr Institute/Usher Institute<br>Frank, John; University of Edinburgh, Farr Institute/Usher Institute
Keywords:	Child Abuse, Epidemiology, Child Psychology

SCHOLARONE™
Manuscripts

What factors are associated with Adverse Childhood Experiences in Scottish children: results from a birth cohort study

Louise Marryat[1], BA (hons.), MA, Ph.D and John Frank[1], B.Sc. (Hon), M.Sc, M.D. (Honours), C.C.F.P., F.R.C.P.

Affiliations: [1] Farr Institute/ Scottish Collaboration for Public Health Research and Policy, University of Edinburgh, UK

Address correspondence to: Louise Marryat, SCPHRP, University of Edinburgh, 20 West Richmond Street, Edinburgh, EH8 9DX, UK louise.marryat@ed.ac.uk , 0131 651 1688.

Short title: Prevalence of ACEs in Scottish children

Funding source: LM is supported by the Farr Institute @ Scotland, which is supported by a 10-funder consortium: Arthritis Research UK, the British Heart Foundation, Cancer Research UK, the Economic and Social Research Council, the Engineering and Physical Sciences Research Council, the Medical Research Council, the National Institute of Health Research, the National Institute for Social Care and Health Research (Welsh Assembly Government), the Chief Scientist Office (Scottish Government Health Directorates), (MRC Grant No: MR/K007017/1). LM sits within, and JF is supported by, the core grant to SCPHRP from the MRC, with half that support from the Scottish Chief Scientist Office (MR/K023209/1)

Conflicts of interest: The authors have no conflicts of interest to declare.

Financial Disclosure: The authors have indicated they have no financial relationships relevant to this article to disclose.

Abbreviations:

- ACEs – Adverse Childhood Experiences
- DWP – department for Work and Pensions
- GUS – Growing Up in Scotland study
- PAR – population attributable risk
- SIMD – Scottish Index of Multiple Deprivation

Table of contents summary: This paper explores Adverse Childhood Experiences (ACEs) using prospective cohort data from Scotland. Using rich cohort data, we investigate which factors are associated with ACEs.

What is known about the subject

Adverse Childhood Experiences (ACEs) have been found to be commonly reported across adult populations. They have been associated with poorer mental and physical health, risky lifestyle behaviours and poorer social and employment outcomes.

Confidential: For Review Only

What this study adds

This study gives an insight into prospectively collected ACEs in a recent cohort. At age 8, children had higher levels of one ACE, but few experienced 4+. ACEs were associated with being male, low income, younger mothers and urban areas.

Contributors statement: Dr Marryat designed the study, cleaned and analysed the data and drafted the final report. Prof. Frank advised on study design and analyses. Dr Marryat and Prof. Frank both read and agreed the final manuscript and agree to be accountable for all aspects of the work.

Confidential: For Review Only

Abstract

Background and Objectives

Adverse childhood experiences (ACEs) have been associated with a range of poorer health and social outcomes throughout the life course, however, to date they have primarily been conducted retrospectively in adulthood. This paper sets out to determine the prevalence of ACEs at age eight in a recent prospective birth cohort, and examine associations between risk factors in the first year and cumulative ACEs.

Design

Children born in Scotland in 2004/5 were identified using Child Benefit Records and followed up for 7 years (n.3119). ACE scores and sample characteristics were calculated and described. Logistic regression models were fitted to explore associations between risk factors and ACE scores. The population attributable risk was calculated for the contribution of poverty to ACE scores.

Results

Two-thirds of children had experienced 1+ ACE, with 10% experiencing 3+. Higher ACE scores were associated with being male, having a young mother, low income, and urban areas. Calculations suggested that removing children from poverty may reduce the proportion of children experiencing 1+ ACEs by 22%.

Conclusions

The majority of children experienced at least one ACE by age eight. ACEs were highly correlated with socio-economic disadvantage in the first year of life.

Confidential: For Review Only

INTRODUCTION

Adverse Childhood Experiences (ACES) were first explored among US adults, and asked a series of questions about childhood experiences covering psychological, physical and sexual abuse, as well as household dysfunction. More than half (52.1%) reported experiencing at least one item[1]. Evidence from England and Wales, respectively, showed similar results [2 3]. A study from New Zealand exploring ACEs in the 1970's suggested that c.58% in a prospective study and c.65% in a retrospective study experienced at least one ACE [4].

Living in adverse socio-economic circumstances during childhood has a demonstrated association with later physical and mental health outcomes [5-7]. ACEs go beyond this to look at other adverse experiences, such as abuse and parental imprisonment. Whilst there is likely to be a substantial overlap with material deprivation, this is generally unknown. The original ACEs study included adults with private health insurance, suggesting that, as adults, this group were relatively affluent [1].

ACEs have been linked to adverse outcomes in childhood and adulthood [1 2 8 9]: they have been associated with poorer self-rated health, premature mortality, suicide attempts, depression, ischemic heart disease, cancer, chronic lung disease, skeletal fractures, liver disease, fetal death, and Chronic Obstructive Pulmonary Disease[1 9-13]. ACEs have also been associated with risky lifestyle behaviours, e.g. increased drug and alcohol use, smoking, and high numbers of sexual partners [1], as well as job, financial, and reproductive problems[11]. They have also been linked to multimorbidity in adulthood [1].

Despite the current interest in ACEs and their seeming importance in relation to subsequent outcomes, we know little about modern experiences of ACEs. The capturing of ACEs in the general population is difficult, and relies on retrospective data collected in adulthood. The aims of this paper are to explore to what extent ACEs could be imputed using prospective cohort data,

Confidential: For Review Only

and what prevalence levels look like in a recent population. This approach allows greater exploration of the predictors of ACEs themselves, normally not available in the retrospective data.

METHOD

Data were taken from the first seven waves of the Growing Up in Scotland Study, covering the first eight years of the child's life. The sample is stratified and clustered, and was derived from child benefit records, which at the time of sampling included 97% of the Scottish population with children. Data zones (the key small-area geographic statistic in Scotland, each containing 500–1000 people) were aggregated until each area had an average of 57 live births per year, based on the previous three years of data, which was estimated to provide the required sample size. These Primary Sampling Units were then stratified by Local Authority (n.32) and then by Scottish Index of Multiple Deprivation Score (a measure of relative area level deprivation taking into account a range of factors). Prior to final sampling, the Department of Work and Pensions removed any 'sensitive cases' (c.5%). Where children were found not to be living with their natural parent, they were followed up where possible in foster care or kinship care. Sweep 1 took place in 2005/6 when the children were 10 months old with 5,217 children, comprising 81% of the eligible children[14]. At Sweep 7 there were 3,456 children (66% of sweep 1 children). The target interview date was at 94.5 months old[15]. Cases were only included were the participant had the relevant ACE and demographic data at each sweep, of which there were 3119 (90.2% of all Sweep 7 subjects). Cases without full data were not used: no imputation was used.

Data from GUS were mapped onto the ACE questions (Supplementary Table 1) and socio-demographic information from the first sweep were linked together. Since ACE-related questionnaire items were asked inconsistently through the seven sweeps of the study, an 8-year cumulative incidence of ACEs was derived by summing the ACEs present for each child throughout the study period. Seven ACEs (or proxies for them) were available. ACEs 1 and 5 were not available. ACE 3 (sexual abuse) was included in an open ended question, however

Confidential: For Review Only

personal communications with the survey team revealed that fewer than 5 participants had responded positively to this, and it was thus excluded.

Ethics

Each sweep of data collection was subject to medical ethical review by the Scotland 'A' MREC committee. All parents interviewed (and Children, for the sweep 7 child interview) gave their informed consent prior to inclusion in the study. The current analyses were subject to ethical review in the University of Edinburgh.

Analyses

Binary variables of 1+ ACE (vs. none) and 3+ACEs (vs. <3) were derived. Data were described and Spearman correlations explored. All correlations were of a weak to medium strength.

Multiple univariable logistic regression models were fitted predicting the risk of having 1+ and 3+ACEs, respectively. Predictors were taken from the current literature: sex, ethnicity, income, age of mother at the birth of the first child and mother's educational qualifications. Where univariable associations reached the criterion of $p<0.1$ these variables were put into the multivariable model and then removed one by one, until the final model was built. It was checked for degree of fit with the r^2 statistic.

To ascertain the impact of poverty on the population of children experiencing ACEs, the population attributable risk (PAR) was calculated[16]. The PAR is the "proportion of the health outcome in an entire population, which is attributable to the exposure" assuming a direct causal relationship. Relative poverty was used as an indicator of those in poverty, using the cut-off of 60% of median income in the population, which in this case equated to £204.88 per week.

The calculation is as follows[16]:

$$x = \frac{\mathrm{Pe}(\mathrm{RRe} - 1)}{1 + \mathrm{Pe}\ (\mathrm{RRe} - 1)}$$

Confidential: For Review Only

RR (relative risk) = proportion of those exposed to poverty who had ACEs/proportion of those unexposed to poverty who had ACEs.
P = proportion of the population who were exposed to poverty.

Results

Two thirds (65%) of children experienced 1+ ACE, with 10% experiencing 3+ at age eight (Figure 1).

[FIGURE 1 ABOUT HERE]

The most common ACEs were parental mental health problems (35.4%), and having parents who were separated or divorced (32.1%). The proportion reporting frequent physical punishment was high (22.2%). Few children experienced parental imprisonment (0.4%).

ACEs varied by household income quintiles: 1% of children in households in the top income quintile had 4+ACEs, compared with 10.8% in the lowest income group (Figure 2).

[FIGURE 2 ABOUT HERE]

Boys were more likely to experience 3+ ACEs (Table 1). Similar patterns could be seen for children from a White UK background, compared with other ethnic groups, although the low proportion (4.3%) of children from non-White-UK backgrounds limited the power of this comparison. Children in urban areas were more likely to have experienced a higher number of ACEs (3.4% vs 1.3% in rural areas).

Confidential: For Review Only

Table 1 Proportion of children in each ACE category by demographics

	Sample size	No. of ACES (%) None	1	2	3	4 or more	*Chi sq. p value*
Sex							
Male	*1609*	32.9	34.6	20.4	8.4	3.8	*0.004*
Female	*1510*	37.3	35.1	18.8	6.5	2.2	
Ethnicity							
White	*2982*	34.7	34.7	19.9	7.6	3.1	*0.07*
Non-white	*135*	43.6	36.7	13.4	5.6	0.6	
Education of mother							
Degree or higher	*842*	51.4	32.9	11.8	2.8	1.1	*<0.001*
Vocational qualification	*1163*	35	35.9	19.2	7.9	2	
Highers	*240*	36.4	32.2	24.9	5.9	0.6	
Standard Grade	*555*	18.7	38.2	23.3	12.3	7.4	
No qualifications	*291*	15.6	32.5	33.3	11.9	6.7	
Other	*24*	56.3	30.7	8	5	0	
Age of mother at birth of 1st child							
Under 20	*541*	12.5	30.1	31.5	16.3	9.5	*<0.001*
20 to 29	*1590*	34.8	35.9	20.1	7.0	2.3	
30 to 39	*945*	48.3	36.2	11.4	3.5	0.6	
40 or over	*35*	46.4	28.8	20.2	2.4	2.2	
Household equivalised income quintile							
Bottom (£<8,410)	*623*	8	27	34.5	19.7	10.8	*<0.001*
2nd quintile (£8,410-£13,749)	*587*	26.6	33.6	26.1	10.6	3.2	
3rd quintile (£13,750-£21,784)	*518*	33.8	38.6	18.3	8.3	1	
4th quintile (£21,785-£33,570)	*592*	42.2	36	17.1	4.2	0.5	
Top quintile (£>=£33,571)	*513*	52.8	32.7	11.1	2.3	1	
Scottish Index of Multiple deprivation (SIMD) area quintile							
1 – Most deprived	*745*	17.2	37.6	29.1	10.9	5.3	*<0.001*
2	*583*	30.3	35.9	19.2	10.9	3.7	
3	*637*	34.6	35.3	22	5.6	2.5	
4	*593*	43	35.2	14.6	5.3	2.0	
5 – Least Deprived	*561*	55.7	29.3	10.2	3.8	1.0	
Urban/rural classification							
Urban	*2567*	32.6	34.9	20.8	8.3	3.4	*<0.001*
Rural	*552*	46.3	34.5	14.3	3.6	1.3	

Independent associations were explored between risk factors in the first year of life and having 1+, or 3+ACEs, respectively. The multivariable model indicated that risk factors predicting having 1+ ACE at age eight were living in a lower income group, in particular living in the lowest income quintile (Odds Ratio 7.11);being male; having a mother with lower educational qualifications; having a mother who was under 20 at the birth of her first

Confidential: For Review Only

child (having a mother aged over 40 was also associated with heightened risk); living in an area with higher levels of deprivation; and living in an urban area. The model explained c.23% of the variation in outcomes (Table 2).

The model predicting having 3+ACEs at age 8 was very similar. The main difference was that, in the multivariable model, maternal educational qualifications were not statistically significant. Once again, the strongest predictor of experiencing 3+ACEs was living in a household in the lowest income quintile, where odds were 6.5 times higher (Table 2). This model explained c.18% of the variation in outcomes.

Confidential: For Review Only

Table 2 Multivariable logistic regression model (with complex samples module) predicting having 1+, and 3+, ACEs, respectively, at age 8.

	**Predicting having 1+ ACE**			**Predicting having 3+ ACEs**		
	Odds ratio	***95% confidence interval***	***P value***	***Odds ratio***	***95% confidence interval***	***P value***
Sex of child						
Male	1.31	1.09-1.56	0.004	1.52	1.15-2.01	0.004
Female	-	-		-	-	
Education of mother			0.001			*NS*
Degree or higher	0.87	0.54-1.41				
Vocational qualification	1.07	0.67-1.73				
Highers	0.94	0.55-1.63				
Standard Grade	1.72	1.07-2.78				
Other	0.41	0.12-1.45				
No qualifications	-	-				
Age of mother at birth of child			<0.001			<0.001
Under 20	1.44	0.69-3.01		2.11	0.44-10.14	
20 to 29	0.75	0.37-1.52		0.98	0.22-4.46	
30 to 39	0.74	0.35-1.59		0.64	0.14-2.91	
40 or over	-	-		-	-	
Household equivalised income quintile			<0.001			<0.001
Bottom (£<8,410)	7.11	4.53-11.17		6.45	3.59-11.61	
2nd quintile (£8,410-£13,749)	1.96	1.52-2.53		2.66	1.38-5.16	
3rd quintile (£13,750-£21,784)	1.52	1.17-1.97		2.31	1.27-4.23	
4th quintile (£21,785-£33,570)	1.38	1.07-1.79		1.32	0.73-2.38	
Top quintile (£>=£33,571)	-	-		-	-	
Scottish Index of Multiple deprivation (SIMD) area quintile			<0.001			*NS*
1 – Most deprived	2.29	1.71-3.06				
2	1.47	1.07-2.01				
3	1.83	1.42-2.36				
4	1.52	1.15-2.02				
5 – Least Deprived	-	-				
Urban/rural classification			<0.001			0.008
Urban	1.57	1.26-1.95		1.82	1.17-2.83	
Rural	-	-		-	-	
R^2	0.23			0.18		
Base	2848			2848		

Confidential: For Review Only

As poverty appeared to play such an important role in children's likelihood of experiencing ACEs, we calculated the population attributable risk (PAR), indicating 'the proportion of the health outcome in an entire population, which is attributable to the exposure'[16] assuming a direct causal relationship. The exposure was living in relative poverty (60% of median household income): 27.6% of the GUS sample lived in relative poverty. In 22% of cases where children experience 1+ ACE, this experience could be directly "attributed" to poverty. On this basis, eliminating child poverty would decrease the proportion of children who experienced 1+ ACE at age eight, from 65%, as it currently stands, to 51%.

Discussion

By eight years, two-thirds of children had experienced 1+ ACE, with one in ten children experiencing 3+. Results indicate that children living in more disadvantaged circumstances were more likely to experience ACEs than their more privileged peers. If childhood poverty was eradicated, and its relationship to ACEs were causal, there would be a 22% reduction in children experiencing 1+ ACEs.

Strengths

This paper is unique in using a range of prospective data on ACEs. GUS covers a representative sample of children from Scotland, providing large enough numbers to explore ACEs by subgroups, combining a mixture of parental and child-reported data, giving a rounded picture of ACEs which have occurred. Response rates were relatively high at each sweep, far higher than the English and Welsh ACE studies, where around half of respondents agreed to participate[2 3]. The inclusion of family background data allows us insight into what early predictors are associated with risk of ACEs. In addition, families were asked to answer a range of questions about their lives, rather than a specific questionnaire about ACEs: Felitti et al. found that respondents to the ACE questionnaire were slightly more likely to have

Confidential: For Review Only

reported sexual abuse in a separate questionnaire, suggesting that there may be a bias within the original ACE studies towards people who had experienced ACEs[1].

Weaknesses

The original ACE questionnaire has not yet been included in GUS and so some proxy information had to be used, which may not accurately reflect the ACE questionnaire. Despite the sample being representative of the population of children in Scotland, some particularly sensitive cases were removed prior to sampling by the DWP, which likely include children who suffered severe adversity within the first 8-9 months. GUS suffers from differential attrition, whereby children from the most disadvantaged backgrounds are disproportionately lost to follow-up[17]. Longitudinal weights do make-up for this to some extent. Two ACEs (emotional abuse and neglect) were not able to be assessed through the GUS questions. In addition, the proportion of parents reporting sexual abuse was so low that we were not able to include this. It is possible that social desirability may lead to some parents not disclosing where ACEs had occurred and, furthermore, if ACEs, such as abuse, happened outside the home, parents may not be aware that they have occurred. All these factors are likely to lead to this study underestimating the prevalence of ACEs, as well as the strength of the associations between poverty and ACEs. From a statistical point of view, many of the risk factors examined had high prevalence rates in this cohort, which may bias away from the null the observed odds ratios produced in the logistic regression models, compared to actual relative risks[18].

The proportion of children with 1+ ACE was substantially higher than most other retrospective studies, in contrast to findings from New Zealand[1-4]. There are various factors which might explain this difference. The first is around recall: data in the latter studies were collected up to forty years after the events took place, compared to continuous collection in GUS. Hardt and Rutter concluded that reports of ACEs in adulthood involve a 'substantial

Confidential: For Review Only

rate of false negatives' due to a lack of very early childhood memory, mood congruent recall biases, and the fact that people are only able to recall what they were aware of at the time[19] – e.g. some parents keep the incarceration of a parent from their children[20].

Cohort effects may also play a role: retrospective studies look back at childhoods between the 1950s and 1970s, where parenting and societal norms often differed from the present day: harsh corporal punishment was far more frequent historically, and still varies substantially between countries [21]. It is noteworthy that the New Zealand cohort with the most recent data (from childhoods in the 1970s-80s) displayed the most similar results to the GUS study[4]. Access to data on cohort differences in ACEs within studies is somewhat limited, although one retrospective study, which looked at four cohorts between 1900 and 1978, found evidence that the proportion of children with no ACEs reported increased from 16.7% to 21.6% over that time -- although those reporting 4+ ACEs also increased slightly, from 43.1% to 51.1%[8].

Children in the GUS sample were less likely to have experienced 3+ ACEs, than the US, New Zealand, Welsh and English studies[1-4]. This may well be due to the difference in age-period examined, where previous studies examined ACEs up to the age of 18, compared with up to the age of eight in GUS, providing less time for children to 'accumulate' ACEs.

Other factors which were associated with having an increased risk of having at least one ACE, or 3+ACEs, respectively, were being male, having a younger mother at the birth of the first child and living in an urban area. The finding that boys were more likely to experience ACEs is based on similar odds ratios to those seen in the previous English retrospective study[23], but was the opposite of findings from Felitti et al., which found that 18% of women experienced 3+ ACEs, compared with 9% of men[1]. The relationship to age of mother was reflected in previous findings: Bellis reported increased odds of higher numbers of ACEs for

Confidential: For Review Only

children born to mothers aged under 20 (not accounting for parity)[23], though these data were not reported in other studies. The exploration of urban/rural differences in ACE counts appears to be a novel contribution to the literature, with no previous evidence produced on these differences, although living in an urban area is independently associated with higher levels of child abuse[24], drug abuse[25] and heavy alcohol use[26] – all contained within the ACE questions.

Further research is needed to find out how many Scottish children experience further ACEs up to age 18, and how this compares to other parts of the world.

Conclusions

Two-thirds of Scottish eight-year-olds had experienced at least one ACE. Whilst this compares unfavourably to previous ACE studies, measurement differences make it difficult to directly compare – although the ACEs reported in this study are clearly a subset of those reported in most studies. Although a large proportion of children had experienced one ACE, just 10% experienced 3+. ACEs were associated with poverty: children living in the lowest household income quintile had odds around seven times higher of having 1+ ACE, than the most affluent children.

Confidential: For Review Only

References

1. Felitti Md FVJ, Anda Md MSRF, Nordenberg Md D, et al. Relationship of childhood abuse and household dysfunction to many of the leading causes of death in adults: The Adverse Childhood Experiences (ACE) Study. American Journal of Preventive Medicine 1998;**14**(4):245-58.
2. Allen MD, A. The impact of adverse experiences in the home on the health of children and young people, and inequalities in the prevalence and effects: UCL Institute of Health Equity, 2015.
3. Ashton K, Bellis M, Hughes K. Adverse childhood experiences and their association with health-harming behaviours and mental wellbeing in the Welsh adult population: a national cross-sectional survey. The Lancet 2016;**388, Supplement 2**:S21.
4. Reuben, Aaron, et al. Lest we forget: comparing retrospective and prospective assessments of adverse childhood experiences in the prediction of adult health. Journal of Child Psychology and Psychiatry 57.10 (2016): 1103-1112.
5. Gershoff ET, Aber JL, Raver CC, et al. Income is not enough: incorporating material hardship into models of income associations with parenting and child development. Child Development 2007;**78**(1):70-95.
6. Danese A, Moffitt TE, Harrington H, et al. Adverse childhood experiences and adult risk factors for age-related disease: Depression, inflammation, and clustering of metabolic risk markers. Archives of Pediatrics & Adolescent Medicine 2009;**163**(12):1135-43.
7. Costello E, Compton SN, Keeler G, et al. Relationships between poverty and psychopathology: A natural experiment. JAMA 2003;**290**(15):2023-29.
8. Dube SR, Felitti VJ, Dong M, et al. The impact of adverse childhood experiences on health problems: evidence from four birth cohorts dating back to 1900. Preventive Medicine 2003;**37**(3):268-77.
9. Chapman DP, Whitfield CL, Felitti VJ, et al. Adverse childhood experiences and the risk of depressive disorders in adulthood. Journal of Affective Disorders 2004;**82**(2):217-25.
10. Dube SR, Anda RF, Felitti VJ, et al. Childhood abuse, household dysfunction, and the risk of attempted suicide throughout the life span: Findings from the adverse childhood experiences study. JAMA 2001;**286**(24):3089-96.
11. Hillis SD, Anda RF, Dube SR, et al. The association between adverse childhood experiences and adolescent pregnancy, long-term psychosocial consequences, and fetal death. Pediatrics 2004;**113**(2):320-27.
12. Brown DW, Anda RF, Tiemeier H, et al. adverse childhood experiences and the risk of premature mortality. American Journal of Preventive Medicine 2009;**37**(5):389-96.
13. Anda RF, Brown DW, Dube SR, et al. adverse childhood experiences and chronic obstructive pulmonary disease in adults. American Journal of Preventive Medicine 2008;**34**(5):396-403.
14. Anderson S, Bradshaw P, Cunningham-Burley S, et al. Growing Up in Scotland: A study following the lives of Scotland's children. 2007.
15. Research SS. Growing Up in Scotland Sweep 7: User Guide. Edinburgh, 2013.
16. Frank J, Jepson R, Williams AJ. *Disease Prevention: A Critical Toolkit*. New York: Oxford University Press, 2016: 28-29.
17. Wolke D, Waylen A, Samara M, et al. Selective drop-out in longitudinal studies and non-biased prediction of behaviour disorders. The British Journal of Psychiatry 2009;**195**(3):249-56.
18. Anderson SA, Ariane; Hauck, Walter W.; Oakes, David; Vandaele, Walter; Weisberg, Herbert I. *Statistical Methods for Comparative Studies*. Canada: Wiley, 1980.

Confidential: For Review Only

19. Hardt J, Rutter M. Validity of adult retrospective reports of adverse childhood experiences: review of the evidence. Journal of Child Psychology and Psychiatry 2004;**45**(2):260-73.
20. Smith PS. *When the Innocent are Punished: Prison, Society and the Effects of Imprisonment. When the Innocent are Punished: The Children of Imprisoned Parents*. London: Palgrave Macmillan UK, 2014:227-33.
21. Straus MA, Mathur AK. Social change and the trends in approval of corporal punishment by parents from 1968 to 1994. Prevention And Intervention In Childhood And Adolescence 1996:91-106.
22. Van Parijs P. Basic income: a simple and powerful idea for the twenty-first century. Politics & Society 2004;**32**(1):7-39.
23. Bellis MA, Lowey H, Leckenby N, et al. Adverse childhood experiences: retrospective study to determine their impact on adult health behaviours and health outcomes in a UK population. Journal of Public Health 2014;**36**(1):81-91.
24. Antai D, Braithwaite P, Clerk G. Social determinants of child abuse: evidence of factors associated with maternal abuse from the Egypt demographic and health survey. Journal of Injury and Violence Research 2016;**8**(1):25-34.
25. McCall-Hosenfeld JS, Mukherjee S, Lehman EB. The prevalence and correlates of lifetime psychiatric disorders and trauma exposures in urban and rural settings: results from the national comorbidity survey replication (NCS-R). PloS one 2014;**9**(11):e112416.
26. Harhay MO, Bor J, Basu S, et al. Differential impact of the economic recession on alcohol use among white British adults, 2004–2010. The European Journal of Public Health 2014;**24**(3):410-15.

Confidential: For Review Only

Figure 1 Number of ACEs experienced in at age eight

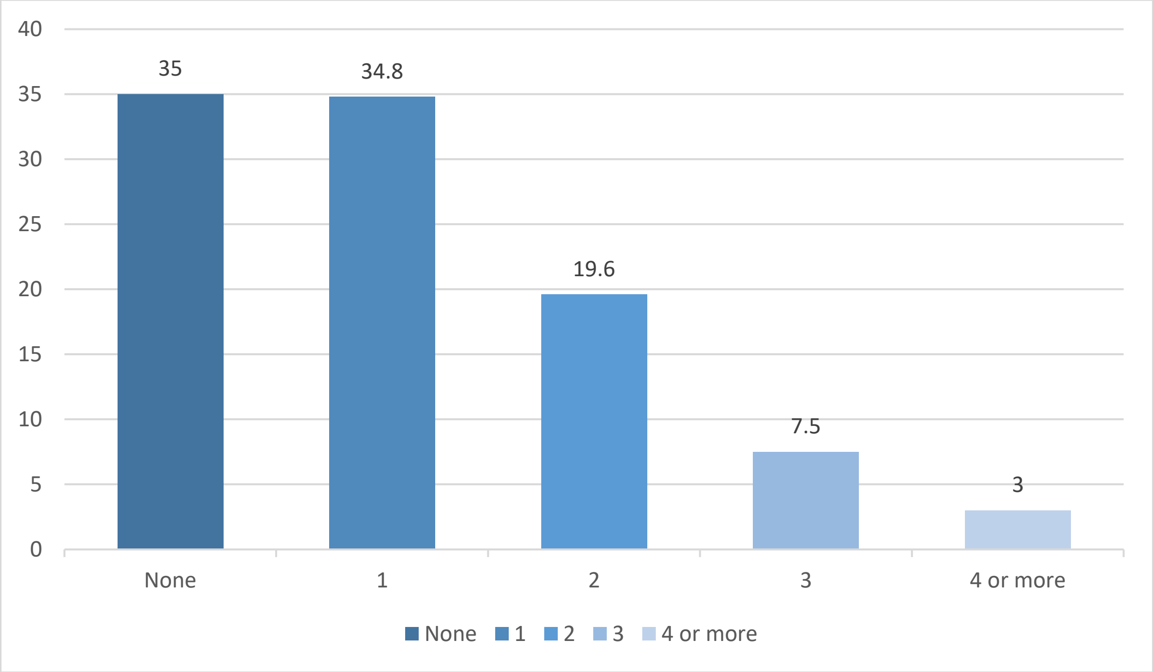

Base: 3119;

Figure 2 Numbers of ACEs experienced by equivalised income quintile at birth

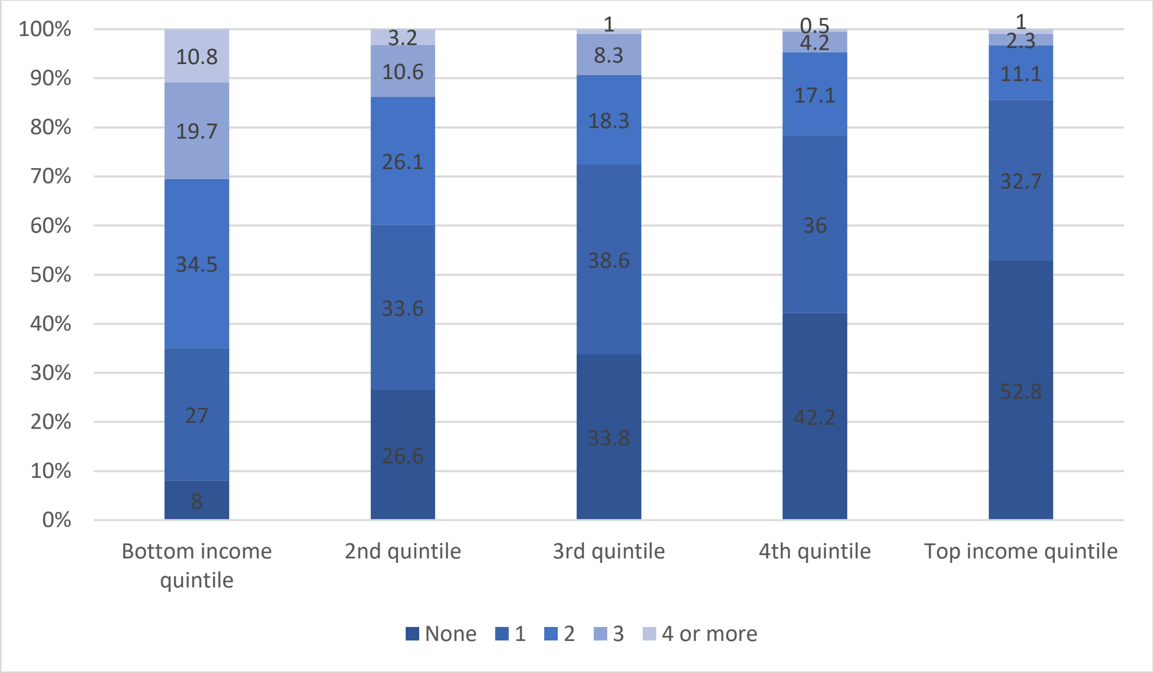

Base: 2832 GUS children with ACEs and income data

Supplemental Table 1 Data availability

**ACE Question**	**Question in GUS**	**Sweep**						
		1	2	3	4	5	6	7
Sociodemographic predictors	Age of mother at birth of first child<br>Equivalised income<br>Education level of mother<br>Household NSSEC<br>Urban/rural classification<br>Scottish Index of Multiple Deprivation (2006)	X<br>X<br>X<br>X<br>X<br>X						
1. Did a parent or other adult in the household **often** …<br><br>Swear at you, insult you, put you down, or humiliate you?<br><br>**Or** Act in a way that made you afraid that you might be physically hurt?	*NOT AVAILABLE*							
2. Did a parent or other adult in the household **often** …<br><br>Push, grab, slap, or throw something at you?<br><br>**Or Ever** hit you so hard that you had marks or were injured?	MGAPQ13 :You slap ^ChildName when ^he has done something wrong – often OR always<br>OR<br>CgPa3 (child int) My parents smack me when I have done something wrong – Often OR always							X<br><br><br>X
3. Did an adult or person at least 5 years older than you **ever**…<br><br>Touch or fondle you or have you touch their body in a sexual way? **Or** Try to or actually have oral, anal, or vaginal sex with you?	*ABUSE WAS COVERED UNDER 'ANY OTHER ADVERSE EVENTS' AT TWO SWEEPS AS AN OPEN ENDED QUESTION, HOWEVER FEWER THAN 5 PARENTS LISTED THIS HAPPENING.*							
4. Did you **often** feel that …<br><br>No one in your family loved you or thought you were important or special?	(child interview)<br>CgPa2: My parents ask about my day in school – NEVER<br>OR<br>CgPa4: My parents play games or do							X

Confidential: For Review Only

**Or** Your family didn't look out for each other, feel close to each other, or support each other?	other fun things with me – NEVER<br>OR<br>CgPa6: My parents help me with my homework – NEVER<br>OR<br>CgPa7: My parents tell me when I'm doing a good job with something- NEVER<br>OR<br>CgPa13: My parents check to make sure I'm doing ok - NEVER							
5. Did you **often** feel that …<br><br>You didn't have enough to eat, had to wear dirty clothes, and had no one to protect you?<br><br>**Or** Your parents were too drunk or high to take care of you or take you to the doctor if you needed it?	*NOT AVAILABLE*							
6. Were your parents **ever** separated or divorced?	If only one natural parent in the household at any sweep (MxHGnp01)	X	X	X	X	X	X	X
7. Was your mother or stepmother:<br><br>**Often** pushed, grabbed, slapped, or had something thrown at her?<br><br>**Or Sometimes or often** kicked, bitten, hit with a fist, or hit with something hard?<br><br>**Or Ever** repeatedly hit over at least a few minutes or threatened with a gun or knife?	IF respondent is mother/stepmother AND IF:<br>And since ^childname was born, has any partner or ex-partner ever done any of these things to you?<br>*MfHdv061* Pushed you or held you down – YES<br>OR<br>*MfHdv062* Kicked, bitten or hit you – YES<br>OR<br>*MfHdv064* Used a weapon against you, for example an ashtray or a bottle – YES<br><br>AND<br><br>*MfHdv07* How many times since ^childname was born have any of these things happened to you? - 3. Four or five times OR 4. Six or more times OR 5. Too many to count<br><br>OR						X	

Confidential: For Review Only

Confidential: For Review Only

	MfHdv034 Threatened you with a weapon, for example an ashtray or a bottle - YES							
8. Did you live with anyone who was a problem drinker or alcoholic or who used street drugs?	Has ^childname experienced any of the things listed on this card since the last interview? *MgOve15* Drug taking/alcoholism in the immediate family - YES<br>Alcohol level >14 units per week (harmful level at time)<br>Use of drugs (use any street drugs in prev 12 months)	X<br>X	X	X<br>X		X<br>X	X	X
9. Was a household member depressed or mentally ill or did a household member attempt suicide?	Has ^childname experienced any of the things listed on this card since the last interview? *MgOve16* Mental disorder in the immediate family – YES<br>OR<br>DASS score of >1SD above 0<br>OR<br>SF12MCS score of <=35	X	X	X	X	X	X	X
10. Did a household member go to prison?	Has ^childname experienced any of the things listed on this card since the last interview? *MgOve20* Parent in prison - YES<br>OR (where available)<br>*MxHGaww3/4*		X			X	X	X

Factors associated with Adverse Childhood Experiences in Scottish children: a prospective cohort study

Journal:	*BMJ Paediatrics Open*
Manuscript ID	bmjpo-2018-000340.R1
Article Type:	Original article
Date Submitted by the Author:	07-Sep-2018
Complete List of Authors:	Marryat, Louise; University of Edinburgh, Farr Institute/Usher Institute<br>Frank, John; University of Edinburgh, Farr Institute/Usher Institute
Keywords:	Child Abuse, Epidemiology, Child Psychology

SCHOLARONE™
Manuscripts

Factors associated with Adverse Childhood Experiences in Scottish children: a prospective cohort study

Louise Marryat[1], BA (hons.), MA, Ph.D and John Frank[1], B.Sc. (Hon), M.Sc, M.D. (Honours), C.C.F.P., F.R.C.P.

Affiliations: [1] Farr Institute/ Scottish Collaboration for Public Health Research and Policy, University of Edinburgh, UK

Address correspondence to: Louise Marryat, SCPHRP, University of Edinburgh, 20 West Richmond Street, Edinburgh, EH8 9DX, UK louise.marryat@ed.ac.uk , 0131 651 1688.

Short title: Prevalence of ACEs in Scottish children

Funding source: LM is supported by the Farr Institute @ Scotland, which is supported by a 10-funder consortium: Arthritis Research UK, the British Heart Foundation, Cancer Research UK, the Economic and Social Research Council, the Engineering and Physical Sciences Research Council, the Medical Research Council, the National Institute of Health Research, the National Institute for Social Care and Health Research (Welsh Assembly Government), the Chief Scientist Office (Scottish Government Health Directorates), (MRC Grant No: MR/K007017/1). LM sits within, and JF is supported by, the core grant to SCPHRP from the MRC, with half that support from the Scottish Chief Scientist Office (MR/K023209/1)

Conflicts of interest: The authors have no conflicts of interest to declare.

Financial Disclosure: The authors have indicated they have no financial relationships relevant to this article to disclose.

Abbreviations:

ACEs – Adverse Childhood Experiences
DWP – department for Work and Pensions
GUS – Growing Up in Scotland study
PAR – population attributable risk
SIMD – Scottish Index of Multiple Deprivation

Table of contents summary: This paper explores Adverse Childhood Experiences (ACEs) using prospective cohort data from Scotland. Using rich cohort data, we investigate which factors are associated with ACEs.

What is known about the subject

Adverse Childhood Experiences (ACEs) have been found to be commonly reported across adult populations. They have been associated with poorer mental and physical health, risky lifestyle behaviours and poorer social and employment outcomes.

Confidential: For Review Only

What this study adds

ACEs were associated with being male, low income, younger mothers and urban areas.

Contributors statement: Dr Marryat designed the study, cleaned and analysed the data and drafted the final report. Prof. Frank advised on study design and analyses. Dr Marryat and Prof. Frank both read and agreed the final manuscript and agree to be accountable for all aspects of the work.

Confidential: For Review Only

Abstract

Background and Objectives

Adverse childhood experiences (ACEs) have been associated with a range of poorer health and social outcomes throughout the life course, however, to date they have primarily been conducted retrospectively in adulthood. This paper sets out to determine the prevalence of ACEs at age eight in a recent prospective birth cohort, and examine associations between risk factors in the first year and cumulative ACEs.

Design

Children born in Scotland in 2004/5 were identified using Child Benefit Records and followed up for 7 years (n.3119). ACE scores and sample characteristics were calculated and described. Logistic regression models were fitted to explore associations between risk factors (sex, mother's age and education, household income, area level deprivation and urban/rural indicator) and ACE scores.

Results

Around two-thirds of children had experienced one or more ACE, with 10% experiencing three or more in their lifetime. Higher ACE scores were associated with being male, having a young mother, low income, and urban areas.

Conclusions

Using prospective data, the majority of children born in 2004/5 in Scotland experienced at least one ACE by age eight, although three of the ten potential ACEs couple not be assessed in this cohort. ACEs were highly correlated with socio-economic disadvantage in the first year of life.

Confidential: For Review Only

INTRODUCTION

Adverse Childhood Experiences (ACEs) were first explored among US adults, who were asked a series of questions covering childhood psychological, physical and sexual abuse, and household dysfunction. Around half (52.1%) reported experiencing at least one item[1]. Evidence from England and Wales, respectively, showed similar results [2 3]. A study from New Zealand exploring ACEs in the 1970's suggested that c.58% in a prospective study and c.65% in a retrospective study experienced at least one ACE [4].

Living in adverse socio-economic circumstances during childhood has a demonstrated association with later physical and mental health outcomes [5-7]. ACEs go beyond this to look at other adversities, e.g. abuse and parental imprisonment. Whilst there is likely to be a substantial overlap with deprivation, this is generally unknown. The original ACE study included only adults with private health insurance, suggesting that, as adults, this group were relatively affluent [1]. Neighbourhood deprivation has been associated with increased levels of ACEs[8], however Bellis et al. only found deprivation to be associated with having four or more ACEs[9]. Higher levels of ACEs have been associated with having a younger mother[9], whilst lower levels have been found among older people, white or Asian people, and graduates[1].

ACEs have been linked to adverse outcomes in childhood and adulthood [1 2 10 11]: they have been associated with poorer self-rated health, premature mortality, suicide attempts, depression, ischemic heart disease, cancer, chronic lung disease, skeletal fractures, liver disease, fetal death, and Chronic Obstructive Pulmonary Disease[1 11-15]. ACEs have also been associated with risky lifestyle behaviours, e.g. drug and alcohol use, smoking, and high numbers of sexual partners [1], as well as job, financial, and reproductive problems[13]. They have also been linked to multimorbidity in adulthood [1].

Confidential: For Review Only

Despite the current interest in ACEs and their seeming importance in relation to subsequent outcomes, we know little about modern ACEs. The capturing of ACEs in the general population is difficult, and relies on retrospective data collected in adulthood. The aims of this paper are to explore to what extent ACEs could be determined using prospective cohort data, and what prevalence levels look like in a recent population. This approach allows exploration of the predictors of ACEs, normally not available in retrospective data.

METHOD

Child and parent interview data were taken from the first seven waves of the Growing Up in Scotland (GUS) Study, covering the first eight years of the child's life. This study, funded by the Scottish Government, tracks the lives of children from birth through to their teenage years and beyond, and collects a wide range of information, including cognitive, social, emotional and behavioural development, health and wellbeing, childcare, education, and parenting and social networks. The full design has been reported elsewhere[16]. The sample was derived from child benefit records, which at the time of sampling included 97% of the Scottish population with children. Data zones (the key small-area geographic statistic in Scotland, each containing 500–1000 people and nested within Local Authorities) were aggregated until each area had an average of 57 live births per year, based on the previous three years of data, which was estimated to provide the required sample size. These Primary Sampling Units were then stratified by Local Authority (n.32) and then by Scottish Index of Multiple Deprivation Score (a measure of relative area level deprivation), before 130 points were chosen at random. Prior to final sampling, the Department of Work and Pensions removed any 'sensitive cases' (e.g. where there had been a death in the immediate family) and any cases which had been sampled in the previous 3 years (c.5%). They then sampled all babies within the selected points which met the date of birth criteria. In cases where more than one child met the criteria, one child was selected at random. Where children were found not to be living with their natural parent, they were followed up where possible in foster care or kinship care. Sweep 1 took place in 2005/6 when the children

Confidential: For Review Only

were 10 months old with 5,217 children, comprising 81% of the eligible children[17]. At Sweep 7 3,456 children remained (66% of sweep 1 children), with a target interview date of 94.5 months old[16]. Cases were only included were the participant had the relevant ACE and demographic data, of which there were 3119 (90.2% of all Sweep 7 subjects). Exploratory analyses indicated that respondents missing data at this stage were more likely to be younger mothers, those living in more deprived areas, and those in urban areas. Cases without full data were not used: no imputation was used. Longitudinal weights, produced by the survey team at ScotCen were used, and the stratification of the sample was accounted for using the Complex Samples module within SPSS24[18]. Full information on weighting can be found in the data user guide[16.]

Data from GUS were mapped onto the ACE questions (Supplementary Table 1) and socio-demographic information from the first sweep were combined into one dataset. Since ACE-related questionnaire items were asked inconsistently throughout the seven sweeps of the study, an 8-year cumulative incidence of ACEs was derived by summing the ACEs present for each child throughout the study period. Seven ACEs (or proxies for them), out of a potential ten, were available. Information on Emotional abuse) and Neglect was not available. Sexual abuse was reported in response to an open-ended question, however personal communications with the survey team revealed that fewer than five participants had responded positively to this, and it was thus excluded.

Ethics

Each sweep of data collection was subject to medical ethical review by the Scotland 'A' MREC committee. All parents interviewed (and Children, for the child interview) gave their informed consent prior to inclusion in the study. The current analyses were subject to ethical review in the University of Edinburgh.

Confidential: For Review Only

Analyses

Binary variables of one or more ACE (vs. none) (1+ ACE) and three or more ACEs (vs. <3) (3+ ACEs) were derived. Three or more ACEs were explored, rather than four or more, due to the number of ACEs assessed being fewer and children having had less time to accumulate them. Socio-demographic data were described and Spearman correlations between them were explored. All correlations were of a weak to medium strength (Supplementary Table 2).

Multiple univariable logistic regression models were fitted predicting the odds of having 1+ and 3+ACEs, respectively (Supplementary Tables 3 and 4). Predictors were taken from the current literature: sex, ethnicity, income, age of mother at the birth of the first child and mother's educational qualifications. Where univariable associations reached the criterion of $p<0.1$ these variables were put into the multivariable model and then removed one by one, until the final model was built.

Results

Around two thirds (65%) of children experienced 1+ ACE, with 10% experiencing 3+ at age eight (Figure 1).

[FIGURE 1 ABOUT HERE]

The most common ACEs were parental mental health problems (35.4%), and having parents who were separated or divorced (32.1%). The proportion reporting frequent physical punishment was high (22.2%). Few children experienced parental imprisonment (0.4%) (Supplementary Table 3).

ACEs varied by household income quintiles: 1% of children in households in the top income quintile had 4+ACEs, compared with 10.8% in the lowest income group (Table 1).

Confidential: For Review Only

173 Boys were more likely to experience 3+ ACEs, as were those with younger mothers, mothers with fewer educational qualifications, and children living in more deprived areas. Similar patterns could be seen for children from a White UK background, compared with other ethnic groups, although the low proportion (4.3%) of children from non-White-UK backgrounds limited the power of this comparison. Children in urban areas were more likely to have experienced a higher number of ACEs (3.4% vs 1.3% in rural areas).

Confidential: For Review Only

Table 1 Proportion of children in each ACE category by demographics

	Sample size	No. of ACES (%) None	1	2	3	4 or more	*Chi sq. p value*
Sex							
Male	*1609*	32.9	34.6	20.4	8.4	3.8	*0.004*
Female	*1510*	37.3	35.1	18.8	6.5	2.2	
Ethnicity							
White	*2982*	34.7	34.7	19.9	7.6	3.1	*0.07*
Non-white	*135*	43.6	36.7	13.4	5.6	0.6	
Education of mother							
Degree or higher	*842*	51.4	32.9	11.8	2.8	1.1	*<0.001*
Vocational qualification	*1163*	35	35.9	19.2	7.9	2	
Highers	*240*	36.4	32.2	24.9	5.9	0.6	
Standard Grade	*555*	18.7	38.2	23.3	12.3	7.4	
No qualifications	*291*	15.6	32.5	33.3	11.9	6.7	
Other	*24*	56.3	30.7	8	5	0	
Age of mother at birth of 1st child							
Under 20	*541*	12.5	30.1	31.5	16.3	9.5	*<0.001*
20 to 29	*1590*	34.8	35.9	20.1	7.0	2.3	
30 to 39	*945*	48.3	36.2	11.4	3.5	0.6	
40 or over	*35*	46.4	28.8	20.2	2.4	2.2	
Household equivalised income quintile							
Bottom (£<8,410)	*623*	8	27	34.5	19.7	10.8	*<0.001*
2nd quintile (£8,410-£13,749)	*587*	26.6	33.6	26.1	10.6	3.2	
3rd quintile (£13,750-£21,784)	*518*	33.8	38.6	18.3	8.3	1	
4th quintile (£21,785-£33,570)	*592*	42.2	36	17.1	4.2	0.5	
Top quintile (£>=£33,571)	*513*	52.8	32.7	11.1	2.3	1	
Scottish Index of Multiple deprivation (SIMD) area quintile							
1 – Most deprived	*745*	17.2	37.6	29.1	10.9	5.3	*<0.001*
2	*583*	30.3	35.9	19.2	10.9	3.7	
3	*637*	34.6	35.3	22	5.6	2.5	
4	*593*	43	35.2	14.6	5.3	2.0	
5 – Least Deprived	*561*	55.7	29.3	10.2	3.8	1.0	
Urban/rural classification							
Urban	*2567*	32.6	34.9	20.8	8.3	3.4	*<0.001*
Rural	*552*	46.3	34.5	14.3	3.6	1.3	

Independent associations were explored between risk factors in the first year of life and having 1+, or 3+ACEs, respectively. The multivariable model indicated that risk factors predicting having 1+ ACE at age eight were living in a lower income group, in particular living in the lowest income quintile (Odds Ratio 7.11);being male; having a mother with lower educational qualifications; having a mother who was under 20 or over 40 at the birth of

Confidential: For Review Only

her first child; living in an area with higher levels of deprivation; or in an urban area. The model explained c.23% of the variation in outcomes (Table 2).

The model predicting having 3+ACEs at age 8 was very similar. The main difference was that, in the multivariable model, maternal educational qualifications were not statistically significant. Once again, the strongest predictor of experiencing 3+ACEs was living in a household in the lowest income quintile, where odds were 5.7 times higher (Table 2). This model explained c.18% of the variation in outcomes.

Confidential: For Review Only

Table 2 Multivariable logistic regression model predicting having 1+, and 3+, ACEs, respectively, at age 8.

	Predicting having 1+ ACE			Predicting having 3+ ACEs		
	Odds ratio	***95% confidence interval***	***P value***	***Odds ratio***	***95% confidence interval***	***P value***
Sex of child						
Male	1.31	1.09-1.56	0.004	1.51	1.15-1.98	0.004
Female	-	-		-	-	
Education of mother			0.001			0.05
Degree or higher	0.87	0.54-1.41		0.86	0.38-1.92	
Vocational qualification	1.07	0.67-1.73		1.10	0.58-2.10	
Highers	0.94	0.55-1.63		0.43	0.16-1.16	
Standard Grade	1.72	1.07-2.78		1.56	0.87-2.78	
Other	0.41	0.12-1.45		0.54	0.05-6.53	
No qualifications	-	-				
Age of mother at birth of child			<0.001			<0.001
Under 20	1.44	0.69-3.01		2.19	0.45-10.57	
20 to 29	0.75	0.37-1.52		1.06	0.23-4.82	
30 to 39	0.74	0.35-1.59		0.70	0.15-3.25	
40 or over	-	-		-	-	
Household equivalised income quintile			<0.001			<0.001
Bottom (£<8,410)	7.11	4.53-11.17		5.73	2.82-11.64	
2nd quintile (£8,410-£13,749)	1.96	1.52-2.53		2.45	1.15-5.20	
3rd quintile (£13,750-£21,784)	1.52	1.17-1.97		2.26	1.17-4.35	
4th quintile (£21,785-£33,570)	1.38	1.07-1.79		1.31	0.71-2.41	
Top quintile (£>=£33,571)	-	-		-	-	
Scottish Index of Multiple deprivation (SIMD) area quintile			<0.001			*0.87*
1 – Most deprived	2.29	1.71-3.06		1.05	0.60-1.84	
2	1.47	1.07-2.01		1.19	0.66-2.16	
3	1.83	1.42-2.36		0.98	0.54-1.80	
4	1.52	1.15-2.02		1.22	0.73-2.04	
5 – Least Deprived	-	-				
Urban/rural classification			<0.001			0.01
Urban	1.57	1.26-1.95		1.82	1.14-2.92	
Rural	-	-		-	-	
Pseudo-R^2	*0.23*			*0.19*		
Sample size	*2848*			*2848*		

Confidential: For Review Only

Discussion

By eight years, around two-thirds of children had 1+ ACE, with one in ten children experiencing 3+. Results indicate that children living in more disadvantaged circumstances were more likely to experience ACEs than their more privileged peers.

Strengths

This paper is unique in using a range of prospective data on ACEs in a current generation of children. GUS covers a representative sample of children from Scotland, providing large enough numbers to explore ACEs by subgroups, combining a mixture of parental and child-reported data, giving a rounded picture of ACEs which have occurred. Response rates were relatively high at each sweep, higher than the English and Welsh general population ACE studies, where around half of respondents agreed to participate[2 3], and similar to the original study of adults within a health insurance scheme (68%)[1]. The inclusion of family background data allows us insight into what early predictors are associated with having higher odds of experiencing ACEs. In addition, families were asked to answer a range of questions about their lives, rather than a specific questionnaire about ACEs: Felitti et al. found that respondents to the ACE questionnaire were slightly more likely to have reported sexual abuse in a separate questionnaire, suggesting that there may be a bias within the original ACE studies towards people who had experienced ACEs[1].

Weaknesses

The original ACE questionnaire has not yet been included in GUS and so some proxy information had to be used, which may not accurately reflect the ACE questionnaire. Two ACEs (emotional abuse and neglect) were not able to be assessed through the GUS questions. In addition, the proportion of parents reporting sexual abuse was so low that we were not able to include this. It is possible that social desirability may lead to some parents not disclosing where ACEs had occurred and, furthermore, if ACEs, such as abuse, happened outside the

Confidential: For Review Only

home, parents may not be aware that they have occurred. All these factors are likely to lead to this study underestimating the prevalence of ACEs. Despite the sample being representative of the population of children in Scotland, some particularly sensitive cases were removed prior to sampling by the DWP, which likely include children who suffered adversity within the first 8-9 months. GUS suffers from differential attrition, whereby children from the most disadvantaged backgrounds are disproportionately lost to follow-up[19]. Longitudinal weights do make-up for this to some extent. From a statistical point of view, many of the risk factors examined had high prevalence rates in this cohort, which may bias away from the null the observed odds ratios produced in the logistic regression models, compared to actual relative risks[20].

The proportion of children with 1+ ACE was substantially higher than most other retrospective studies, in contrast to findings from New Zealand[1-4]. There are various factors which might explain this difference. Differences in the way questions are phrased and proxies used are likely to affect prevalence reported. Secondly, there are issues around recall: data in the latter studies were collected up to forty years after the events took place, compared to continuous collection in GUS. Hardt and Rutter concluded that reports of ACEs in adulthood involve a 'substantial rate of false negatives' due to a lack of very early childhood memory, mood congruent recall biases, and the fact that people are only able to recall what they were aware of at the time[21] – e.g. some parents keep the incarceration of a parent from their children[22].

Cohort effects may also play a role: retrospective studies look back at childhoods between the 1950s and 1970s, where parenting and societal norms often differed from the present day: harsh corporal punishment was far more frequent historically, and still varies substantially between countries[23]. It is noteworthy that the New Zealand cohort with the most recent data (from childhoods in the 1970s-80s) displayed the most similar results to the GUS study[4].

Confidential: For Review Only

Access to data on cohort differences in ACEs within studies is somewhat limited, although one retrospective study, which looked at four cohorts between 1900 and 1978, found evidence that the proportion of children with no ACEs reported increased from 16.7% to 21.6% over that time -- although those reporting 4+ ACEs also increased slightly, from 43.1% to 51.1%[8].

Children in the GUS sample were less likely to have experienced 3+ ACEs, than the US, New Zealand, Welsh and English studies[1-4]. This may well be due to the difference in age-period examined, where previous studies examined ACEs up to the age of 18, compared with up to the age of eight in GUS, providing less time for children to 'accumulate' ACEs.

Other factors which were associated with having increased odds of having 1+ ACE, or 3+ACEs, respectively, were being male, having a younger mother and living in an urban area. The finding that boys were more likely to experience ACEs is similar to that seen in the previous English retrospective study[2], but was the opposite of findings from Felitti et al., which found that 18% of women experienced 3+ ACEs, compared with 9% of men[1]. The relationship to age of mother was reflected in previous findings: Bellis reported increased odds of higher numbers of ACEs for children born to mothers aged under 20 [9]. The exploration of urban/rural differences in ACE counts appears to be a novel contribution to the literature, with no previous evidence produced on these differences, although living in an urban area is independently associated with higher levels of child abuse[24], drug abuse[25] and heavy alcohol use[26] – all contained within the ACE questions.A substantial proportion of variation within the current models remained unexplained: future research may wish to explore the explanatory power of factors such as attachment[27], neurodevelopmental disorders[28], parenting and parental ACEs[29-30]. Additional research is also needed to find out how many Scottish children experience further ACEs up to age 18, and how this compares to other parts of the world.

Confidential: For Review Only

Conclusions

Around two-thirds of Scottish eight-year-olds had experienced at least one ACE during their life. Whilst this compares unfavourably to previous ACE studies, measurement differences make it difficult to directly compare – although the ACEs reported in this study are clearly a subset of those reported in most studies. Although a large proportion of children had experienced one ACE, just 10% experienced 3+. ACEs were associated with poverty: children living in the lowest household income quintile had odds around seven times higher of having 1+ ACE, than the most affluent children.

Confidential: For Review Only

References

1. Felitti Md FVJ, Anda Md MSRF, Nordenberg Md D, et al. Relationship of childhood abuse and household dysfunction to many of the leading causes of death in adults: The Adverse Childhood Experiences (ACE) Study. American Journal of Preventive Medicine 1998;**14**(4):245-58.

2. Allen MD, A. The impact of adverse experiences in the home on the health of children and young people, and inequalities in the prevalence and effects: UCL Institute of Health Equity, 2015.

3. Ashton K, Bellis M, Hughes K. Adverse childhood experiences and their association with health-harming behaviours and mental wellbeing in the Welsh adult population: a national cross-sectional survey. The Lancet 2016;**388, Supplement 2**:S21.

4. Reuben, Aaron, et al. Lest we forget: comparing retrospective and prospective assessments of adverse childhood experiences in the prediction of adult health. Journal of Child Psychology and Psychiatry 57.10 (2016): 1103-1112.

5. Gershoff ET, Aber JL, Raver CC, et al. Income is not enough: incorporating material hardship into models of income associations with parenting and child development. Child Development 2007;**78**(1):70-95.

6. Danese A, Moffitt TE, Harrington H, et al. Adverse childhood experiences and adult risk factors for age-related disease: Depression, inflammation, and clustering of metabolic risk markers. Archives of Pediatrics & Adolescent Medicine 2009;**163**(12):1135-43.

7. Costello E, Compton SN, Keeler G, et al. Relationships between poverty and psychopathology: A natural experiment. JAMA 2003;**290**(15):2023-29.

8. Baglivio MT, Wolff KT, Epps N, Nelson R. Predicting adverse childhood experiences: The importance of neighborhood context in youth trauma among delinquent youth. Crime & Delinquency. 2017 Feb;63(2):166-88.

9. Bellis MA, Lowey H, Leckenby N, et al. Adverse childhood experiences: retrospective study to determine their impact on adult health behaviours and health outcomes in a UK population. Journal of Public Health 2014;36(1):81-91.

10. Dube SR, Felitti VJ, Dong M, et al. The impact of adverse childhood experiences on health problems: evidence from four birth cohorts dating back to 1900. Preventive Medicine 2003;**37**(3):268-77.

11. Chapman DP, Whitfield CL, Felitti VJ, et al. Adverse childhood experiences and the risk of depressive disorders in adulthood. Journal of Affective Disorders 2004;**82**(2):217-25.

12. Dube SR, Anda RF, Felitti VJ, et al. Childhood abuse, household dysfunction, and the risk of attempted suicide throughout the life span: Findings from the adverse childhood experiences study. JAMA 2001;**286**(24):3089-96.

13. Hillis SD, Anda RF, Dube SR, et al. The association between adverse childhood experiences and adolescent pregnancy, long-term psychosocial consequences, and fetal death. Pediatrics 2004;**113**(2):320-27.

14. Brown DW, Anda RF, Tiemeier H, et al. adverse childhood experiences and the risk of premature mortality. American Journal of Preventive Medicine 2009;**37**(5):389-96.

15. Anda RF, Brown DW, Dube SR, et al. adverse childhood experiences and chronic obstructive pulmonary disease in adults. American Journal of Preventive Medicine 2008;**34**(5):396-403.

16. Research SS. Growing Up in Scotland Sweep 7: User Guide. Edinburgh, 2013.

17. Anderson S, Bradshaw P, Cunningham-Burley S, et al. Growing Up in Scotland: A study following the lives of Scotland's children. 2007.

18. IBM Corp. Released 2016. IBM SPSS Statistics for Windows, Version 24.0. Armonk, NY: IBM Corp.

Confidential: For Review Only

19. Wolke D, Waylen A, Samara M, et al. Selective drop-out in longitudinal studies and non-biased prediction of behaviour disorders. The British Journal of Psychiatry 2009;**195**(3):249-56.

20. Anderson SA, Ariane; Hauck, Walter W.; Oakes, David; Vandaele, Walter; Weisberg, Herbert I. *Statistical Methods for Comparative Studies*. Canada: Wiley, 1980.

21. Hardt J, Rutter M. Validity of adult retrospective reports of adverse childhood experiences: review of the evidence. Journal of Child Psychology and Psychiatry 2004;**45**(2):260-73.

22. Smith PS. *When the Innocent are Punished: Prison, Society and the Effects of Imprisonment. When the Innocent are Punished: The Children of Imprisoned Parents*. London: Palgrave Macmillan UK, 2014:227-33.

23. Straus MA, Mathur AK. Social change and the trends in approval of corporal punishment by parents from 1968 to 1994. Prevention And Intervention In Childhood And Adolescence 1996:91-106.

24. Antai D, Braithwaite P, Clerk G. Social determinants of child abuse: evidence of factors associated with maternal abuse from the Egypt demographic and health survey. Journal of Injury and Violence Research 2016;**8**(1):25-34.

25. McCall-Hosenfeld JS, Mukherjee S, Lehman EB. The prevalence and correlates of lifetime psychiatric disorders and trauma exposures in urban and rural settings: results from the national comorbidity survey replication (NCS-R). PloS one 2014;**9**(11):e112416.

26. Harhay MO, Bor J, Basu S, et al. Differential impact of the economic recession on alcohol use among white British adults, 2004–2010. The European Journal of Public Health 2014;**24**(3):410-15.

27. Rutter M. Resilience in the face of adversity: Protective factors and resistance to psychiatric disorder. The British Journal of Psychiatry. 1985 Dec;147(6):598-611.

28. Dinkler L, Lundström S, Gajwani R, Lichtenstein P, Gillberg C, Minnis H. Maltreatment□associated neurodevelopmental disorders: a co□twin control analysis. Journal of Child Psychology and Psychiatry. 2017 Jun;58(6):691-701.

29. Zuravin S, McMillen C, DePanfilis D, Risley-Curtiss C. The intergenerational cycle of child maltreatment: Continuity versus discontinuity. Journal of interpersonal violence. 1996 Sep;11(3):315-34.

30. Narayan AJ, Kalstabakken AW, Labella MH, Nerenberg LS, Monn AR, Masten AS. Intergenerational continuity of adverse childhood experiences in homeless families: Unpacking exposure to maltreatment versus family dysfunction. American Journal of Orthopsychiatry. 2017;87(1):3.

Confidential: For Review Only

Figure 1 Number of ACEs reported at age eight

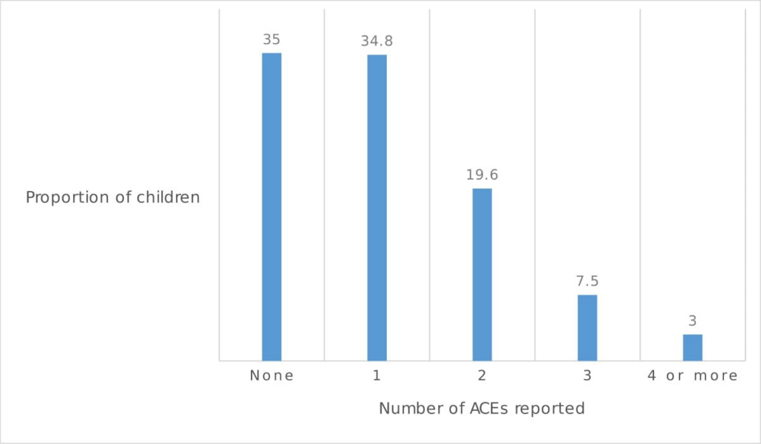

Sample size: 3119

416x410mm (96 x 96 DPI)

Supplementary Table 1 Source of data used in ACE calculations

ACE Question$	Question in GUS	Respondent	Sweep						
			1	2	3	4	5	6	7
Sociodemographic<br><br>predictors	Age of mother at birth of first child<br>Equivalised income<br>Education level of mother<br>Household National Statistics Socio-economic Classification<br>Urban/rural classification<br>Scottish Index of Multiple Deprivation (2006)	Parent	X<br><br>X<br>X<br>X<br><br>X<br>X						
1. Did a parent or other adult in the household **often** …<br><br>Swear at you, insult you, put you down, or humiliate you?<br><br>**Or** Act in a way that made you afraid that you might be physically hurt?	*NOT AVAILABLE*	N/A							
2. Did a parent or other adult in the household **often** …<br><br>Push, grab, slap, or throw something at you?<br><br>**Or Ever** hit you so hard that you had marks or were injured?	MGAPQ13 :You slap ^ChildName when ^he has done something wrong – often OR always<br>OR<br>CgPa3 (child int) My parents smack me when I have done something wrong – Often OR always	Parent<br><br><br><br><br>Child							X<br><br><br>X
3. Did an adult or person at least 5 years older than you **ever**…<br><br>Touch or fondle you or have you touch	*ABUSE WAS COVERED UNDER 'ANY OTHER ADVERSE EVENTS' AT TWO SWEEPS AS AN OPEN ENDED QUESTION, HOWEVER FEWER THAN 5 PARENTS LISTED THIS HAPPENING.*	Parent							

Confidential: For Review Only

their body in a sexual way? **Or** Try to or actually have oral, anal, or vaginal sex with you?									
4. Did you **often** feel that …<br><br>No one in your family loved you or thought you were important or special?<br><br>**Or** Your family didn't look out for each other, feel close to each other, or support each other?	(child interview)<br>CgPa2: My parents ask about my day in school – NEVER<br>OR<br>CgPa4: My parents play games or do other fun things with me – NEVER<br>OR<br>CgPa6: My parents help me with my homework – NEVER<br>OR<br>CgPa7: My parents tell me when I'm doing a good job with something- NEVER<br>OR<br>CgPa13: My parents check to make sure I'm doing ok - NEVER	Child							X
5. Did you **often** feel that …<br><br>You didn't have enough to eat, had to wear dirty clothes, and had no one to protect you?<br><br>**Or** Your parents were too drunk or high to take care of you or take you to the doctor if you needed it?	*NOT AVAILABLE*	N/A							
6. Were your parents **ever** separated or divorced?	If only one natural parent in the household at any sweep (MxHGnp01)	Parent	X	X	X	X	X	X	X
7. Was your mother or stepmother:<br><br>**Often** pushed,	IF respondent is mother/stepmother AND IF: And since ^childname was born, has any partner or ex-partner ever done any of	Parent						X	

Confidential: For Review Only

grabbed, slapped, or had something thrown at her?<br><br>**Or Sometimes or often** kicked, bitten, hit with a fist, or hit with something hard?<br><br>**Or Ever** repeatedly hit over at least a few minutes or threatened with a gun or knife?	these things to you?<br>*MfHdv061* Pushed you or held you down – YES<br>OR<br>*MfHdv062* Kicked, bitten or hit you – YES<br>OR<br>*MfHdv064* Used a weapon against you, for example an ashtray or a bottle – YES<br><br>AND<br><br>*MfHdv07* How many times since ^childname was born have any of these things happened to you? - 3. Four or five times OR 4. Six or more times OR 5. Too many to count<br><br>OR<br><br>*MfHdv034* Threatened you with a weapon, for example an ashtray or a bottle - YES								
8. Did you live with anyone who was a problem drinker or alcoholic or who used street drugs?	Has ^childname experienced any of the things listed on this card since the last interview?<br>*MgOve15* Drug taking/alcoholism in the immediate family - YES<br>Alcohol level >14 units per week (harmful level at time)<br>Use of drugs (use any street drugs in prev 12 months)	Parent	X<br>X	X	X<br>X		X<br>X	X	X
9. Was a household member depressed or mentally ill or did a household member attempt suicide?	Has ^childname experienced any of the things listed on this card since the last interview?<br>*MgOve16* Mental disorder in the immediate family – YES<br>OR<br>DASS* score of >1SD above 0<br>OR<br>SF12MCS** score of <=35	Parent	X	X	X	X	X	X	X
10. Did a household member go to prison?	Has ^childname experienced any of the things listed on this card since the last interview?<br>*MgOve20* Parent in prison - YES	Parent		X			X	X	X

Confidential: For Review Only

	OR (where available)<br>*MxHGaww3/4*									

*DASS: Depression, Anxiety and Stress Scale
**SF12MCS: Short Form 12 Mental Component Scale
$These are the same 10 ACEs used in the comprehensive Wave 2 of the original ACE Study (Dong et al., 2004).

Confidential: For Review Only

Supplementary Table 3 Spearman correlations between socio-demographic data

	1.	2.	3.	4.	5.	6.
1. Sex	1	0.03*	0.01	-0.01	-0.01	<0.01
2. Age of mother at birth of child		1	0.50**	-0.42**	-0.36**	0.04
3. Household income quintiles			1	-0.51**	-0.49**	0.04*
4. Respondent education				1	0.38**	-0.06**
5. Scottish Index of Multiple Deprivation Quintiles					1	-0.18**
6. Urban-rural classification						1

*correlation is significant at the 0.05 level
** correlation is significant at the 0.01 level

r_s	*Interpretation*
0.00-0.19	Very weak
0.20-0.39	Weak
0.40-0.59	Moderate
0.60-0.79	Strong
0.80-1.0	Very strong

Confidential: For Review Only

Supplementary Table 3 Univariable logistic regression models predicting having one or more ACE at age 8

	Sample size	Odds ratio	95% confidence interval	R^2	P value
Sex				0.003	0.03
Male	*1609*	1.21	1.03-1.44		
Female	*1510*	-	-		
Ethnicity					
White	*2982*	1.46	0.95-2.48	0.002	0.10
Non-white	*135*	-	-		
Education of mother				0.1	<0.001
Degree or higher	*842*	0.18	0.11-0.27		
Vocational qualification	*1163*	0.34	0.22-0.53		
Highers	*240*	0.32	0.20-0.53		
Standard Grade	*555*	0.80	0.51-1.27		
Other	*24*	0.14	0.05-0.43		
No qualifications	*291*	-	-		
Age of mother at birth of 1st child				0.09	<0.001
Under 20	*541*	6.07	3.16-11.66		
20 to 29	*1590*	1.62	0.87-3.02		
30 to 39	*945*	0.93	0.49-1.75		
40 or over	*35*	-	-		
Household equivalised income quintile				0.18	<0.001
Bottom (£<8,410)	*623*	15.05	9.67-23.43		
2nd quintile (£8,410-£13,749)	*586*	3.16	2.36-4.22		
3rd quintile (£13,750-£21,784)	*519*	2.07	1.59-2.69		
4th quintile (£21,785-£33,570)	*592*	1.59	1.23-2.05		
Top quintile (£>=£33,571)	*513*	-	-		
Scottish Index of Multiple deprivation (SIMD) area quintile				0.1	<0.001
1 – Most deprived	*745*	6.06	4.71-7.80		
2	*583*	2.90	2.25-3.73		
3	*637*	2.38	1.93-2.94		
4	*593*	1.67	1.31-2.13		
5 – Least Deprived	*561*	-	-		
Urban/rural classification				0.02	<0.001
Urban	*2567*	1.79	1.50-2.13		
Rural	*552*	-	-		

Confidential: For Review Only

Supplementary Table 4 Univariable logistic regression models predicting having three or more ACE at age 8

	Sample size	Odds ratio	95% confidence interval	R^2	P value
Sex				0.007	0.01
Male	*1609*	1.45	1.11-1.89		
Female	*1510*	1.00			
Ethnicity				0.002	0.15
White	*2982*	1.80	0.80-4.03		
Non-white	*135*	1.00			
Education of mother					
Degree or higher	*842*	0.18	0.10-0.33	0.07	<0.001
Vocational qualification	*1163*	0.48	0.30-0.79		
Highers	*240*	0.30	0.13-0.67		
Standard Grade	*555*	1.08	0.67-1.73		
Other	*24*	0.23	0.03-1.85		
No qualifications	*291*	1.00			
Age of mother at birth of 1st child				0.10	<0.001
Under 20	*541*	7.26	1.58-33.37		
20 to 29	*1590*	2.12	0.49-9.16		
30 to 39	*945*	0.88	0.20-3.88		
40 or over	*35*	1.00			
Household equivalised income quintile				0.14	<0.001
Bottom (£<8,410)	*623*	11.99	7.02-20.47		
2nd quintile (£8,410-£13,749)	*586*	3.90	2.12-7.16		
3rd quintile (£13,750-£21,784)	*519*	2.97	1.70-5.20		
4th quintile (£21,785-£33,570)	*592*	1.46	0.80-2.65		
Top quintile (£>=£33,571)	*513*	1.00			
Scottish Index of Multiple deprivation (SIMD) area quintile				0.04	<0.001
1 – Most deprived	*745*	1.56	0.89-2.72		
2	*583*	1.75	1.00-3.05		
3	*637*	3.51	2.02-5.75		
4	*593*	3.84	2.33-6.34		
5 – Least Deprived	*561*	1.00			
Urban/rural classification				0.02	<0.001
Urban	*2567*	2.56	1.62-4.05		
Rural	*552*	1.00			

Confidential: For Review Only

Supplementary Table 5 Proportion of children meeting each criteria

**ACE field**	**Weighted percentage**
ACE1: emotional abuse	-
ACE2: corporal punishment	22.2%
ACE3: sexual abuse	-
ACE4: not loved or supported	20%
ACE5: neglect	-
ACE6: Parents ever divorced or separated	32.1%
ACE7: Domestic violence	9.1%
ACE8: Drug or alcohol misuse	14.0%
ACE9: Mental health problems	35.4%
ACE10: Parent in prison	0.4%
Any ACE reported	65.0%
Base	*3119*

Confidential: For Review Only

Factors associated with Adverse Childhood Experiences in Scottish children: a prospective cohort study

Journal:	*BMJ Paediatrics Open*
Manuscript ID	bmjpo-2018-000340.R2
Article Type:	Original article
Date Submitted by the Author:	18-Oct-2018
Complete List of Authors:	Marryat, Louise; University of Edinburgh, Farr Institute/Usher Institute; University of Edinburgh Centre for Clinical Brain Sciences, Salvesen Mindroom Centre<br>Frank, John; University of Edinburgh, Farr Institute/Usher Institute
Keywords:	Child Abuse, Epidemiology, Child Psychology

SCHOLARONE™
Manuscripts

Factors associated with Adverse Childhood Experiences in Scottish children: a prospective cohort study

Louise Marryat[1], BA (hons.), MA, Ph.D and John Frank[1], B.Sc. (Hon), M.Sc, M.D. (Honours), C.C.F.P., F.R.C.P.

Affiliations: [1] Farr Institute/ Scottish Collaboration for Public Health Research and Policy, University of Edinburgh, UK

Address correspondence to: Louise Marryat, SCPHRP, University of Edinburgh, 20 West Richmond Street, Edinburgh, EH8 9DX, UK louise.marryat@ed.ac.uk , 0131 651 1688.

Short title: Prevalence of ACEs in Scottish children

Funding source: LM is supported by the Farr Institute @ Scotland, which is supported by a 10-funder consortium: Arthritis Research UK, the British Heart Foundation, Cancer Research UK, the Economic and Social Research Council, the Engineering and Physical Sciences Research Council, the Medical Research Council, the National Institute of Health Research, the National Institute for Social Care and Health Research (Welsh Assembly Government), the Chief Scientist Office (Scottish Government Health Directorates), (MRC Grant No: MR/K007017/1). LM sits within, and JF is supported by, the core grant to SCPHRP from the MRC, with half that support from the Scottish Chief Scientist Office (MR/K023209/1)

Conflicts of interest: The authors have no conflicts of interest to declare.

Financial Disclosure: The authors have indicated they have no financial relationships relevant to this article to disclose.

Abbreviations:

ACEs – Adverse Childhood Experiences
DWP – department for Work and Pensions
GUS – Growing Up in Scotland study
PAR – population attributable risk
SIMD – Scottish Index of Multiple Deprivation

Table of contents summary: This paper explores Adverse Childhood Experiences (ACEs) using prospective cohort data from Scotland. Using rich cohort data, we investigate which factors are associated with ACEs.

What is known about the subject

Adverse Childhood Experiences (ACEs) have been found to be commonly reported across adult populations. Limited evidence suggests that higher levels of ACEs are found among adults who

Confidential: For Review Only

had a younger mother, and who live in more deprived neighbourhoods. Lower levels have been seen in older populations, white or Asian populations and among graduates.

What this study adds

ACEs were associated with being male, low income, younger mothers and urban areas in a current child cohort.

Contributors statement: Dr Marryat designed the study, cleaned and analysed the data and drafted the final report. Prof. Frank advised on study design and analyses. Dr Marryat and Prof. Frank both read and agreed the final manuscript and agree to be accountable for all aspects of the work.

Confidential: For Review Only

Abstract

Background and Objectives

Adverse childhood experiences (ACEs) have been associated with a range of poorer health and social outcomes throughout the life course, however, to date they have primarily been conducted retrospectively in adulthood. This paper sets out to determine the prevalence of ACEs at age eight in a recent prospective birth cohort, and examine associations between risk factors in the first year and cumulative ACEs.

Design

This study uses the Growing Up in Scotland Birth Cohort 1, in which children born in Scotland in 2004/5 were identified using Child Benefit Records, and followed up for 7 years (n.3119). ACE scores and sample characteristics were calculated and described. Logistic regression models were fitted to explore associations between risk factors (sex, mother's age and education, household income, area level deprivation and urban/rural indicator) and ACE scores.

Results

Seven ACEs (or proxies) were assessed: physical abuse, domestic violence, substance abuse, mental illness, parental separation, parental incarceration, and emotional neglect. Instances of sexual abuse were too few to be reported. Emotional abuse and physical neglect could not be gathered. Around two-thirds of children had experienced one or more ACE, with 10% experiencing three or more in their lifetime. Higher ACE scores were associated with being male, having a young mother, low income, and urban areas.

Conclusions

Using prospective data, the majority of children born in 2004/5 in Scotland experienced at least one ACE by age eight, although three ACEs could not be assessed in this cohort. ACEs were highly correlated with socio-economic disadvantage in the first year of life.

Confidential: For Review Only

INTRODUCTION

The Adverse Childhood Experiences (ACEs) scale was first explored with US adults, who were asked a series of questions covering childhood psychological, physical and sexual abuse, and household dysfunction. Around half (52.1%) reported experiencing at least one item[1]. Evidence from England and Wales, respectively, showed similar results [2 3]. A study from New Zealand exploring ACEs in the 1970's suggested that c.58% in a prospective study and c.65% in a retrospective study experienced at least one ACE [4].

Living in adverse socio-economic circumstances during childhood has a demonstrated association with later physical and mental health outcomes [5-7]. ACEs go beyond this to look at other adversities, e.g. abuse and parental imprisonment. Whilst there is likely to be a substantial overlap with deprivation, this is generally unknown, although evidence of associations with individual measures does exist [5 8 9]. The original ACE study included only adults with private health insurance, suggesting that, as adults, this group were relatively affluent [1]. Neighbourhood deprivation has been associated with increased levels of ACEs[10], however Bellis et al. only found deprivation to be associated with having four or more ACEs[11]. Higher levels of ACEs have been associated with having a younger mother[11], whilst lower levels have been found among older people, white or Asian people, and graduates[1].

ACEs have been linked to adverse outcomes in childhood and adulthood [1 2 12 13]: they have been associated with poorer self-rated health, premature mortality, suicide attempts, depression, ischemic heart disease, cancer, chronic lung disease, skeletal fractures, liver disease, fetal death, and Chronic Obstructive Pulmonary Disease[1 13-17]. ACEs have also been associated with risky lifestyle behaviours, e.g. drug and alcohol use, smoking, and high numbers of sexual partners [1],

Confidential: For Review Only

as well as job, financial, and reproductive problems[15]. They have been linked to multimorbidity in adulthood [1].

Despite the current interest in ACEs and their seeming importance in relation to subsequent outcomes, we know little about modern ACEs. The capturing of ACEs in the general population is difficult, and relies on retrospective data collected in adulthood. The aims of this paper are to explore to what extent ACEs could be determined using prospective cohort data, and what prevalence levels look like in a recent population. This approach allows exploration of the predictors of ACEs, normally not available in retrospective data.

METHOD

Child and parent interview data were taken from the first seven waves of the Growing Up in Scotland (GUS) Study, covering the first eight years of the child's life. This study, funded by the Scottish Government, tracks the lives of children from birth through to their teenage years and beyond, and collects a wide range of information, including cognitive, social, emotional and behavioural development, health and wellbeing, childcare, education, and parenting and social networks. The full design has been reported elsewhere[18]. The sample was taken from child benefit records, which at the time of sampling included 97% of the Scottish population with children. Data zones (the key small-area geographic statistic in Scotland, each containing 500–1000 people and nested within Local Authorities) were aggregated until each area had an average of 57 live births per year, based on the previous three years of data, which was estimated to provide the required sample size. These Primary Sampling Units were then stratified by Local Authority (n.32) and then by Scottish Index of Multiple Deprivation Score (a measure of relative area level deprivation), before 130 points were chosen at random. Prior to final sampling, the Department of Work and Pensions removed any 'sensitive cases' (e.g. where there had been a death in the immediate family) and any cases which had been sampled

Confidential: For Review Only

in the previous 3 years (c.5%). They then sampled all babies within the selected points which met the date of birth criteria. In cases where more than one child met the criteria, one child was selected at random. Where children were found not to be living with their natural parent, they were followed up where possible in foster care or kinship care. Sweep 1 took place in 2005/6 when the children were 10 months old with 5,217 children, comprising 81% of the eligible children[19]. At Sweep 7 3,456 children remained (66% of sweep 1 children), with a target interview date of 94.5 months old[18]. Supplementary Table 1 details the missing cases. Cases were only included were the participant had the relevant ACE and demographic data, of which there were 3119 (90.2% of all Sweep 7 subjects). Exploratory analyses indicated that respondents missing data at this stage were more likely to be younger mothers, those living in more deprived areas, and those in urban areas. Cases without full data were not used: no imputation was used. Longitudinal weights, produced by the survey team at ScotCen were used, and the stratification of the sample was accounted for using the Complex Samples module within SPSS24[20]. Full information on weighting can be found in the data user guide[18.]

Data from GUS were mapped onto the ACE questions (Supplementary Table 2) and socio-demographic information from the first sweep were combined into one dataset. Since ACE-related questionnaire items were asked inconsistently throughout the seven sweeps of the study, an 8-year cumulative incidence of ACEs was derived by summing the ACEs present for each child throughout the study period. Seven ACEs (or proxies for them), were available: physical abuse, domestic violence, substance abuse, mental illness, parental separation, parental incarceration, and emotional neglect. Information on Emotional abuse and Neglect was not available. Sexual abuse was reported in response to an open-ended question, however personal communications with the survey team revealed that fewer than five participants had responded positively to this, and it was thus excluded.

Confidential: For Review Only

Ethics

Each sweep of data collection was subject to medical ethical review by the Scotland 'A' MREC committee. All parents interviewed (and Children, for the child interview) gave their informed consent prior to inclusion in the study. The current analyses were subject to ethical review in the University of Edinburgh.

Analyses

Binary variables of one or more ACE (vs. none) (1+ ACE) and three or more ACEs (vs. <3) (3+ ACEs) were derived. Three or more ACEs were explored, rather than four or more, due to the number of ACEs assessed being fewer and children having had less time to accumulate them. Socio-demographic data were described and Spearman correlations between them were explored. All correlations were of a weak to medium strength (Supplementary Table 3).

Multiple univariable logistic regression models were fitted predicting the odds of having 1+ and 3+ACEs, respectively (Supplementary Tables 4 and 5). Predictors were taken from the current literature: sex, ethnicity, income, age of mother at the birth of the first child and mother's educational qualifications. Where univariable associations reached the criterion of $p<0.1$ these variables were put into the multivariable model.

Results

Around two thirds (65%) of children experienced 1+ ACE, with 10% experiencing 3+ at age eight (Figure 1).

[FIGURE 1 ABOUT HERE]

Confidential: For Review Only

The most common ACEs were parental mental health problems (35.4%), and having parents who were separated or divorced (32.1%). The proportion reporting frequent physical punishment was high (22.2%). Few children experienced parental imprisonment (0.4%) (Table 1).

Table 1 Proportion of children within each ACE category

ACE field	Weighted percentage
ACE1: emotional abuse	-
ACE2: corporal punishment	22.2%
ACE3: sexual abuse	-
ACE4: not loved or supported	20%
ACE5: neglect	-
ACE6: Parents ever divorced or separated	32.1%
ACE7: Domestic violence	9.1%
ACE8: Drug or alcohol misuse	14.0%
ACE9: Mental health problems	35.4%
ACE10: Parent in prison	0.4%
Any ACE reported	65.0%
Base	*3119*

Confidential: For Review Only

ACEs varied by household income quintiles: 1% of children in households in the top income quintile had 4+ACEs, compared with 10.8% in the lowest income group (Table 2).

Boys were more likely to experience 3+ ACEs, as were those with younger mothers, mothers with fewer educational qualifications, and children living in more deprived areas. A higher

182 proportion of children from a White UK background, compared with other ethnic groups, experienced 3+ ACEs, although the low proportion (4.3%) of children from non-White-UK backgrounds limited the power of this comparison. Children in urban areas were more likely to have experienced a higher number of ACEs (3.4% vs 1.3% in rural areas).

Confidential: For Review Only

Table 2 Proportion of children in each ACE category by demographics

	Sample size	No. of ACES (%) None	1	2	3	4 or more	*Chi sq. p value*
Sex							
Male	*1609*	32.9	34.6	20.4	8.4	3.8	*0.004*
Female	*1510*	37.3	35.1	18.8	6.5	2.2	
Ethnicity							
White	*2982*	34.7	34.7	19.9	7.6	3.1	*0.07*
Non-white	*135*	43.6	36.7	13.4	5.6	0.6	
Education of mother							
Degree or higher	*842*	51.4	32.9	11.8	2.8	1.1	*<0.001*
Vocational qualification	*1163*	35	35.9	19.2	7.9	2	
Highers	*240*	36.4	32.2	24.9	5.9	0.6	
Standard Grade	*555*	18.7	38.2	23.3	12.3	7.4	
No qualifications	*291*	15.6	32.5	33.3	11.9	6.7	
Other	*24*	56.3	30.7	8	5	0	
Age of mother at birth of 1st child							
Under 20	*541*	12.5	30.1	31.5	16.3	9.5	*<0.001*
20 to 29	*1590*	34.8	35.9	20.1	7.0	2.3	
30 to 39	*945*	48.3	36.2	11.4	3.5	0.6	
40 or over	*35*	46.4	28.8	20.2	2.4	2.2	
Household equivalised income quintile							
Bottom (£<8,410)	*623*	8	27	34.5	19.7	10.8	*<0.001*
2nd quintile (£8,410-£13,749)	*587*	26.6	33.6	26.1	10.6	3.2	
3rd quintile (£13,750-£21,784)	*518*	33.8	38.6	18.3	8.3	1	
4th quintile (£21,785-£33,570)	*592*	42.2	36	17.1	4.2	0.5	
Top quintile (£>=£33,571)	*513*	52.8	32.7	11.1	2.3	1	
Scottish Index of Multiple deprivation (SIMD) area quintile							
1 – Most deprived	*745*	17.2	37.6	29.1	10.9	5.3	*<0.001*
2	*583*	30.3	35.9	19.2	10.9	3.7	
3	*637*	34.6	35.3	22	5.6	2.5	
4	*593*	43	35.2	14.6	5.3	2.0	
5 – Least Deprived	*561*	55.7	29.3	10.2	3.8	1.0	
Urban/rural classification							
Urban	*2567*	32.6	34.9	20.8	8.3	3.4	*<0.001*
Rural	*552*	46.3	34.5	14.3	3.6	1.3	

Independent associations were explored between risk factors in the first year of life and having 1+, or 3+ACEs, respectively. The multivariable model indicated that risk factors predicting having 1+ ACE at age eight were living in a lower income group, in particular living in the lowest income quintile (Odds Ratio 7.11);being male; having a mother with lower educational

Confidential: For Review Only

qualifications; having a mother who was under 20 or over 40 at the birth of her first child; living in an area with higher levels of deprivation; or in an urban area. (Table 3).

The model predicting having 3+ACEs at age 8 was very similar. In this multivariable model, maternal educational qualifications did not meet our criteria for statistical significance, although it is worth noting that the odds ratios were similar to the previous model. Once again, the strongest predictor of experiencing 3+ACEs was living in a household in the lowest income quintile, where odds were 5.7 times higher (Table 3).

Confidential: For Review Only

Table 3 Multivariable logistic regression model predicting having 1+, and 3+, ACEs, respectively, at age 8.

	Predicting having 1+ ACE			Predicting having 3+ ACEs		
	Odds ratio	***95% confidence interval***	***P value***	***Odds ratio***	***95% confidence interval***	***P value***
Sex of child						
Male	1.31	1.09-1.56	0.004	1.51	1.15-1.98	0.004
Female	-	-		-	-	
Education of mother			0.001			0.05
Degree or higher	0.87	0.54-1.41		0.86	0.38-1.92	
Vocational qualification	1.07	0.67-1.73		1.10	0.58-2.10	
Highers	0.94	0.55-1.63		0.43	0.16-1.16	
Standard Grade	1.72	1.07-2.78		1.56	0.87-2.78	
Other	0.41	0.12-1.45		0.54	0.05-6.53	
No qualifications	-	-				
Age of mother at birth of child			<0.001			<0.001
Under 20	1.44	0.69-3.01		2.19	0.45-10.57	
20 to 29	0.75	0.37-1.52		1.06	0.23-4.82	
30 to 39	0.74	0.35-1.59		0.70	0.15-3.25	
40 or over	-	-		-	-	
Household equivalised income quintile			<0.001			<0.001
Bottom (£<8,410)	7.11	4.53-11.17		5.73	2.82-11.64	
2nd quintile (£8,410-£13,749)	1.96	1.52-2.53		2.45	1.15-5.20	
3rd quintile (£13,750-£21,784)	1.52	1.17-1.97		2.26	1.17-4.35	
4th quintile (£21,785-£33,570)	1.38	1.07-1.79		1.31	0.71-2.41	
Top quintile (£>=£33,571)	-	-		-	-	
Scottish Index of Multiple deprivation (SIMD) area quintile			<0.001			*0.87*
1 – Most deprived	2.29	1.71-3.06		1.05	0.60-1.84	
2	1.47	1.07-2.01		1.19	0.66-2.16	
3	1.83	1.42-2.36		0.98	0.54-1.80	
4	1.52	1.15-2.02		1.22	0.73-2.04	
5 – Least Deprived	-	-				
Urban/rural classification			<0.001			0.01
Urban	1.57	1.26-1.95		1.82	1.14-2.92	
Rural	-	-		-	-	
Sample size	*2848*			*2848*		

Confidential: For Review Only

Discussion

By eight years, around two-thirds of children had 1+ ACE, with one in ten children experiencing 3+. Results indicate that children living in more disadvantaged circumstances were more likely to experience ACEs than their more privileged peers.

Strengths

This paper is unique in using a range of prospective data on ACEs in a current generation of children. GUS covers a representative sample of children from Scotland, providing large enough numbers to explore ACEs by subgroups, combining a mixture of parental and child-reported data, giving a rounded picture of ACEs which have occurred. The inclusion of family background data allows us insight into what early predictors are associated with having higher odds of experiencing ACEs. In addition, families were asked to answer a range of questions about their lives, rather than a specific questionnaire about ACEs: Felitti et al. found that respondents to the ACE questionnaire were slightly more likely to have reported sexual abuse in a separate questionnaire, suggesting that there may be a bias within the original ACE studies towards people who had experienced ACEs[1].

Weaknesses

The original ACE questionnaire has not yet been included in GUS and so some proxy information had to be used, which may not accurately reflect the ACE questionnaire. Two ACEs (emotional abuse and neglect) were not able to be assessed through the GUS questions. In addition, the proportion of parents reporting sexual abuse was so low that we were not able to include this. It is possible that social desirability may lead to some parents not disclosing where ACEs had occurred and, furthermore, if ACEs, such as abuse, happened outside the home, parents may not be aware that they have occurred. All these factors are likely to lead to this study

Confidential: For Review Only

underestimating the prevalence of ACEs. Despite the sample being representative of the population of children in Scotland, some particularly sensitive cases were removed prior to sampling by the DWP, which likely include children who suffered adversity within the first 8-9 months. GUS suffers from differential attrition, whereby children from the most disadvantaged backgrounds are disproportionately lost to follow-up[21]. Longitudinal weights do make-up for this to some extent.

The proportion of children with 1+ ACE was substantially higher than most other retrospective studies, in contrast to findings from New Zealand[1-4]. There are various factors which might explain this difference. Differences in the way questions are phrased and proxies used are likely to affect prevalence reported. Secondly, there are issues around recall: data in the latter studies were collected up to forty years after the events took place, compared to continuous collection in GUS. Hardt and Rutter concluded that reports of ACEs in adulthood involve a 'substantial rate of false negatives' due to a lack of very early childhood memory, mood congruent recall biases, and the fact that people are only able to recall what they were aware of at the time[22] – e.g. some parents keep the incarceration of a parent from their children[22].

Cohort effects may also play a role: retrospective studies look back at childhoods between the 1950s and 1970s, where parenting and societal norms often differed from the present day: harsh corporal punishment was far more frequent historically, and still varies substantially between countries[8]. It is noteworthy that the New Zealand cohort with the most recent data (from childhoods in the 1970s-80s) displayed the most similar results to the GUS study[4]. Access to data on cohort differences in ACEs within studies is somewhat limited, although one retrospective study, which looked at four cohorts between 1900 and 1978, found evidence that the proportion

Confidential: For Review Only

of children with no ACEs reported increased from 16.7% to 21.6% over that time - although those reporting 4+ ACEs also increased slightly, from 43.1% to 51.1%[12].

Children in the GUS sample were less likely to have experienced 3+ ACEs, than the US, New Zealand, Welsh and English studies[1-4]. This may well be due to the difference in age-period examined, where previous studies examined ACEs up to the age of 18, compared with up to the age of eight in GUS, providing less time for children to 'accumulate' ACEs.

Other factors which were associated with having increased odds of having 1+ ACE, or 3+ACEs, respectively, were being male, having a younger mother and living in an urban area. The finding that boys were more likely to experience ACEs is similar to that seen in the previous English retrospective study[2], but was the opposite of findings from Felitti et al., which found that 18% of women experienced 3+ ACEs, compared with 9% of men. This may be due to the two of the items which were unable to be measured (sexual abuse and emotional abuse), both of which are more likely to be experienced by women[1]. The relationship to age of mother was reflected in previous findings: Bellis reported increased odds of higher numbers of ACEs for children born to mothers aged under 20 [11]. The exploration of urban/rural differences in ACE counts appears to be a novel contribution to the literature, with no previous evidence produced on these differences, although living in an urban area is independently associated with higher levels of child abuse[9], drug abuse[23] and heavy alcohol use[24] – all contained within the ACE questions. These models focussed on socio-demographic risks for ACEs: future research may wish to explore the explanatory power of factors such as attachment[25], neurodevelopmental disorders[26], parenting and parental ACEs[27-28]. Additional research is also needed to find out how many Scottish children experience further ACEs up to age 18, and how this compares to other parts of the world.

Confidential: For Review Only

Conclusions

Around two-thirds of Scottish eight-year-olds had experienced at least one ACE during their life. Whilst this compares unfavourably to previous ACE studies, measurement differences make it difficult to directly compare – although the ACEs reported in this study are clearly a subset of those reported in most studies. Although a large proportion of children had experienced one ACE, just 10% experienced 3+. ACEs were associated with poverty: children living in the lowest household income quintile had odds around seven times higher of having 1+ ACE, than the most affluent children.

Confidential: For Review Only

References

1. Felitti Md FVJ, Anda Md MSRF, Nordenberg Md D, et al. Relationship of childhood abuse and household dysfunction to many of the leading causes of death in adults: The Adverse Childhood Experiences (ACE) Study. American Journal of Preventive Medicine 1998;**14**(4):245-58.
2. Allen MD, A. The impact of adverse experiences in the home on the health of children and young people, and inequalities in the prevalence and effects: UCL Institute of Health Equity, 2015.
3. Ashton K, Bellis M, Hughes K. Adverse childhood experiences and their association with health-harming behaviours and mental wellbeing in the Welsh adult population: a national cross-sectional survey. The Lancet 2016;**388, Supplement 2**:S21.
4. Reuben, Aaron, et al. Lest we forget: comparing retrospective and prospective assessments of adverse childhood experiences in the prediction of adult health. Journal of Child Psychology and Psychiatry 57.10 (2016): 1103-1112.
5. Gershoff ET, Aber JL, Raver CC, et al. Income is not enough: incorporating material hardship into models of income associations with parenting and child development. Child Development 2007;**78**(1):70-95.
6. Danese A, Moffitt TE, Harrington H, et al. Adverse childhood experiences and adult risk factors for age-related disease: Depression, inflammation, and clustering of metabolic risk markers. Archives of Pediatrics & Adolescent Medicine 2009;**163**(12):1135-43.
7. Costello E, Compton SN, Keeler G, et al. Relationships between poverty and psychopathology: A natural experiment. JAMA 2003;**290**(15):2023-29.
8. Straus MA, Mathur AK. Social change and the trends in approval of corporal punishment by parents from 1968 to 1994. Prevention And Intervention In Childhood And Adolescence 1996:91-106.
9. Antai D, Braithwaite P, Clerk G. Social determinants of child abuse: evidence of factors associated with maternal abuse from the Egypt demographic and health survey. Journal of Injury and Violence Research 2016;**8**(1):25-34.
10. Baglivio MT, Wolff KT, Epps N, Nelson R. Predicting adverse childhood experiences: The importance of neighborhood context in youth trauma among delinquent youth. Crime & Delinquency. 2017 Feb;63(2):166-88.
11. Bellis MA, Lowey H, Leckenby N, et al. Adverse childhood experiences: retrospective study to determine their impact on adult health behaviours and health outcomes in a UK population. Journal of Public Health 2014;36(1):81-91.
12. Dube SR, Felitti VJ, Dong M, et al. The impact of adverse childhood experiences on health problems: evidence from four birth cohorts dating back to 1900. Preventive Medicine 2003;**37**(3):268-77.
13. Chapman DP, Whitfield CL, Felitti VJ, et al. Adverse childhood experiences and the risk of depressive disorders in adulthood. Journal of Affective Disorders 2004;**82**(2):217-25.
14. Dube SR, Anda RF, Felitti VJ, et al. Childhood abuse, household dysfunction, and the risk of attempted suicide throughout the life span: Findings from the adverse childhood experiences study. JAMA 2001;**286**(24):3089-96.
15. Hillis SD, Anda RF, Dube SR, et al. The association between adverse childhood experiences and adolescent pregnancy, long-term psychosocial consequences, and fetal death. Pediatrics 2004;**113**(2):320-27.

Confidential: For Review Only

16. Brown DW, Anda RF, Tiemeier H, et al. adverse childhood experiences and the risk of premature mortality. American Journal of Preventive Medicine 2009;**37**(5):389-96.
17. Anda RF, Brown DW, Dube SR, et al. adverse childhood experiences and chronic obstructive pulmonary disease in adults. American Journal of Preventive Medicine 2008;**34**(5):396-403.
18. Research SS. Growing Up in Scotland Sweep 7: User Guide. Edinburgh, 2013.
19. Anderson S, Bradshaw P, Cunningham-Burley S, et al. Growing Up in Scotland: A study following the lives of Scotland's children. 2007.
20. IBM Corp. Released 2016. IBM SPSS Statistics for Windows, Version 24.0. Armonk, NY: IBM Corp.
21. Wolke D, Waylen A, Samara M, et al. Selective drop-out in longitudinal studies and non-biased prediction of behaviour disorders. The British Journal of Psychiatry 2009;**195**(3):249-56.
22. Hardt J, Rutter M. Validity of adult retrospective reports of adverse childhood experiences: review of the evidence. Journal of Child Psychology and Psychiatry 2004;**45**(2):260-73.
23. McCall-Hosenfeld JS, Mukherjee S, Lehman EB. The prevalence and correlates of lifetime psychiatric disorders and trauma exposures in urban and rural settings: results from the national comorbidity survey replication (NCS-R). PloS one 2014;**9**(11):e112416.
24. Harhay MO, Bor J, Basu S, et al. Differential impact of the economic recession on alcohol use among white British adults, 2004–2010. The European Journal of Public Health 2014;**24**(3):410-15.
25. Rutter M. Resilience in the face of adversity: Protective factors and resistance to psychiatric disorder. The British Journal of Psychiatry. 1985 Dec;147(6):598-611.
26. Dinkler L, Lundström S, Gajwani R, Lichtenstein P, Gillberg C, Minnis H. Maltreatment-associated neurodevelopmental disorders: a co-twin control analysis. Journal of Child Psychology and Psychiatry. 2017 Jun;58(6):691-701.
27. Zuravin S, McMillen C, DePanfilis D, Risley-Curtiss C. The intergenerational cycle of child maltreatment: Continuity versus discontinuity. Journal of interpersonal violence. 1996 Sep;11(3):315-34.
38. Narayan AJ, Kalstabakken AW, Labella MH, Nerenberg LS, Monn AR, Masten AS. Intergenerational continuity of adverse childhood experiences in homeless families: Unpacking exposure to maltreatment versus family dysfunction. American Journal of Orthopsychiatry. 2017;87(1):3.

Confidential: For Review Only

Figure 1 Number of ACEs reported at age eight

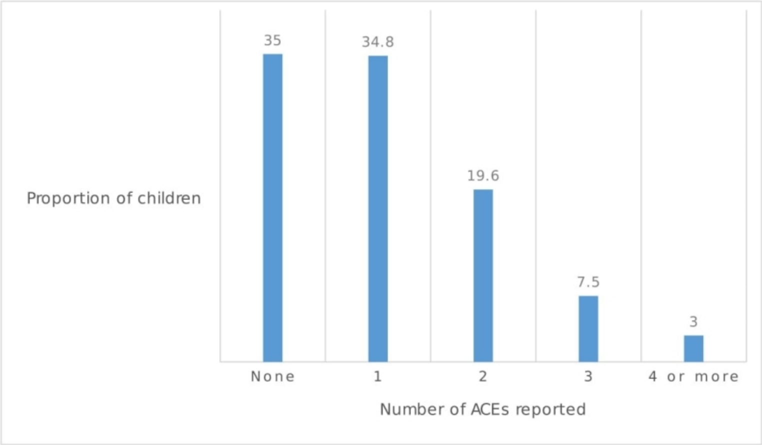

Sample size: 3119

416x410mm (96 x 96 DPI)

Proportion of children within various demographic groups who were in the sample at Sweep 1, but not in the sample at sweep 7

	Missing at sweep 7 (%)
Family type	
Lone parent	53.5
Living with partner	29.2
Age of mother at birth of cohort child	
Under 20	62.6
20-29	41.4
30-39	24.1
40 or older	25.1
Income quintile	
Bottom quintile	50.8
2	36.2
3	28.5
4	24.3
Top quintile	23.5
Education level of mother	
No qualifications	54.6
Standard Grades or above	46.8
Highers or above	27.8
Child ethnic group	
White	33
Non-White	48.5
Child sex	
Male	34.8
Female	32.6
Urban/rural indicator	
Large urban	35.8
Other urban	37.5
Small accessible towns	32.3
Small remote towns	28.2
Accessible rural	25.8
Remote rural	22.3
Base	*1761*

Confidential: For Review Only

Supplementary Table 2 Source of data used in ACE calculations

**ACE Question[$]**	**Question in GUS**	**Respondent**	**Sweep**						
			1	2	3	4	5	6	7
Sociodemographic<br><br>predictors	Age of mother at birth of first child<br>Equivalised income<br>Education level of mother<br>Household National Statistics Socio-economic Classification<br>Urban/rural classification<br>Scottish Index of Multiple Deprivation (2006)	Parent	X<br><br>X<br>X<br>X<br><br>X<br>X						
1. Did a parent or other adult in the household **often** …<br><br>Swear at you, insult you, put you down, or humiliate you?<br><br>**Or** Act in a way that made you afraid that you might be physically hurt?	*NOT AVAILABLE*	N/A							
2. Did a parent or other adult in the household **often** …<br><br>Push, grab, slap, or throw something at you?<br><br>**Or Ever** hit you so hard that you had marks or were injured?	MGAPQ13 :You slap ^ChildName when ^he has done something wrong – often OR always<br>OR<br>CgPa3 (child int) My parents smack me when I have done something wrong – Often OR always	Parent<br><br><br><br>Child							X<br><br><br>X
3. Did an adult or person at least 5	*ABUSE WAS COVERED UNDER 'ANY OTHER ADVERSE EVENTS' AT TWO SWEEPS AS AN OPEN*	Parent							

Confidential: For Review Only

years older than you **ever**…<br><br>Touch or fondle you or have you touch their body in a sexual way? **Or** Try to or actually have oral, anal, or vaginal sex with you?	*ENDED QUESTION, HOWEVER FEWER THAN 5 PARENTS LISTED THIS HAPPENING.*								
4. Did you **often** feel that …<br><br>No one in your family loved you or thought you were important or special?<br><br>**Or** Your family didn't look out for each other, feel close to each other, or support each other?	(child interview)<br>CgPa2: My parents ask about my day in school – NEVER<br>OR<br>CgPa4: My parents play games or do other fun things with me – NEVER<br>OR<br>CgPa6: My parents help me with my homework – NEVER<br>OR<br>CgPa7: My parents tell me when I'm doing a good job with something- NEVER<br>OR<br>CgPa13: My parents check to make sure I'm doing ok - NEVER	Child							X
5. Did you **often** feel that …<br><br>You didn't have enough to eat, had to wear dirty clothes, and had no one to protect you?<br><br>**Or** Your parents were too drunk or high to take care of you or take you to the doctor if you needed it?	*NOT AVAILABLE*	N/A							

Confidential: For Review Only

6. Were your parents **ever** separated or divorced?	If only one natural parent in the household at any sweep (MxHGnp01)	Parent	X	X	X	X	X	X	X
7. Was your mother or stepmother:<br><br>**Often** pushed, grabbed, slapped, or had something thrown at her?<br><br>**Or Sometimes or often** kicked, bitten, hit with a fist, or hit with something hard?<br><br>**Or Ever** repeatedly hit over at least a few minutes or threatened with a gun or knife?	IF respondent is mother/stepmother AND IF: And since ^childname was born, has any partner or ex-partner ever done any of these things to you?<br>*MfHdv061* Pushed you or held you down – YES<br>OR<br>*MfHdv062* Kicked, bitten or hit you – YES<br>OR<br>*MfHdv064* Used a weapon against you, for example an ashtray or a bottle – YES<br><br>AND<br><br>*MfHdv07* How many times since ^childname was born have any of these things happened to you? - 3. Four or five times OR 4. Six or more times OR 5. Too many to count<br><br>OR<br><br>*MfHdv034* Threatened you with a weapon, for example an ashtray or a bottle - YES	Parent						X	
8. Did you live with anyone who was a problem drinker or alcoholic or who used street drugs?	Has ^childname experienced any of the things listed on this card since the last interview?<br>*MgOve15* Drug taking/alcoholism in the immediate family - YES<br>Alcohol level >14 units per week (harmful level at time)<br>Use of drugs (use any street drugs in prev 12 months)	Parent	X<br>X	X	X<br>X		X<br>X	X	X

Confidential: For Review Only

9. Was a household member depressed or mentally ill or did a household member attempt suicide?	Has ^childname experienced any of the things listed on this card since the last interview?<br>*MgOve16* Mental disorder in the immediate family – YES<br>OR<br>DASS* score of >1SD above 0<br>OR<br>SF12MCS** score of <=35	Parent	X	X	X	X	X	X	X
10. Did a household member go to prison?	Has ^childname experienced any of the things listed on this card since the last interview?<br>*MgOve20* Parent in prison - YES<br>OR (where available)<br>*MxHGaww3/4*	Parent		X			X	X	X

*DASS: Depression, Anxiety and Stress Scale
**SF12MCS: Short Form 12 Mental Component Scale
$These are the same 10 ACEs used in the comprehensive Wave 2 of the original ACE Study (Dong et al., 2004).

Confidential: For Review Only

Supplementary Table 3 Spearman correlations between socio-demographic data

	1.	2.	3.	4.	5.	6.
1. Sex	1	0.03*	0.01	-0.01	-0.01	<0.01
2. Age of mother at birth of child		1	0.50**	-0.42**	-0.36**	0.04
3. Household income quintiles			1	-0.51**	-0.49**	0.04*
4. Respondent education				1	0.38**	-0.06**
5. Scottish Index of Multiple Deprivation Quintiles					1	-0.18**
6. Urban-rural classification						1

*correlation is significant at the 0.05 level
** correlation is significant at the 0.01 level

r_s	*Interpretation*
0.00-0.19	Very weak
0.20-0.39	Weak
0.40-0.59	Moderate
0.60-0.79	Strong
0.80-1.0	Very strong

Confidential: For Review Only

Supplementary Table 4 Univariable logistic regression models predicting having one or more ACE at age 8

	Sample size	Odds ratio	95% confidence interval	P value
Sex				0.03
Male	*1609*	1.21	1.03-1.44	
Female	*1510*	-	-	
Ethnicity				0.10
White	*2982*	1.46	0.95-2.48	
Non-white	*135*	-	-	
Education of mother				<0.001
Degree or higher	*842*	0.18	0.11-0.27	
Vocational qualification	*1163*	0.34	0.22-0.53	
Highers	*240*	0.32	0.20-0.53	
Standard Grade	*555*	0.80	0.51-1.27	
Other	*24*	0.14	0.05-0.43	
No qualifications	*291*	-	-	
Age of mother at birth of 1st child				<0.001
Under 20	*541*	6.07	3.16-11.66	
20 to 29	*1590*	1.62	0.87-3.02	
30 to 39	*945*	0.93	0.49-1.75	
40 or over	*35*	-	-	
Household equivalised income quintile				<0.001
Bottom (£<8,410)	*623*	15.05	9.67-23.43	
2nd quintile (£8,410-£13,749)	*586*	3.16	2.36-4.22	
3rd quintile (£13,750-£21,784)	*519*	2.07	1.59-2.69	
4th quintile (£21,785-£33,570)	*592*	1.59	1.23-2.05	
Top quintile (£>=£33,571)	*513*	-	-	
Scottish Index of Multiple deprivation (SIMD) area quintile				<0.001
1 – Most deprived	*745*	6.06	4.71-7.80	
2	*583*	2.90	2.25-3.73	
3	*637*	2.38	1.93-2.94	
4	*593*	1.67	1.31-2.13	
5 – Least Deprived	*561*	-	-	
Urban/rural classification				<0.001
Urban	*2567*	1.79	1.50-2.13	
Rural	*552*	-	-	

Confidential: For Review Only

Supplementary Table 5 Univariable logistic regression models predicting having three or more ACE at age 8

	Sample size	Odds ratio	95% confidence interval	R^2	P value
Sex				0.007	0.01
Male	*1609*	1.45	1.11-1.89		
Female	*1510*	1.00			
Ethnicity				0.002	0.15
White	*2982*	1.80	0.80-4.03		
Non-white	*135*	1.00			
Education of mother					
Degree or higher	*842*	0.18	0.10-0.33	0.07	<0.001
Vocational qualification	*1163*	0.48	0.30-0.79		
Highers	*240*	0.30	0.13-0.67		
Standard Grade	*555*	1.08	0.67-1.73		
Other	*24*	0.23	0.03-1.85		
No qualifications	*291*	1.00			
Age of mother at birth of 1st child				0.10	<0.001
Under 20	*541*	7.26	1.58-33.37		
20 to 29	*1590*	2.12	0.49-9.16		
30 to 39	*945*	0.88	0.20-3.88		
40 or over	*35*	1.00			
Household equivalised income quintile				0.14	<0.001
Bottom (£<8,410)	*623*	11.99	7.02-20.47		
2nd quintile (£8,410-£13,749)	*586*	3.90	2.12-7.16		
3rd quintile (£13,750-£21,784)	*519*	2.97	1.70-5.20		
4th quintile (£21,785-£33,570)	*592*	1.46	0.80-2.65		
Top quintile (£>=£33,571)	*513*	1.00			
Scottish Index of Multiple deprivation (SIMD) area quintile				0.04	<0.001
1 – Most deprived	*745*	1.56	0.89-2.72		
2	*583*	1.75	1.00-3.05		
3	*637*	3.51	2.02-5.75		
4	*593*	3.84	2.33-6.34		
5 – Least Deprived	*561*	1.00			
Urban/rural classification				0.02	<0.001
Urban	*2567*	2.56	1.62-4.05		
Rural	*552*	1.00			

Confidential: For Review Only

Factors associated with Adverse Childhood Experiences in Scottish children: a prospective cohort study

Journal:	*BMJ Paediatrics Open*
Manuscript ID	bmjpo-2018-000340.R3
Article Type:	Original article
Date Submitted by the Author:	20-Nov-2018
Complete List of Authors:	Marryat, Louise; University of Edinburgh, Farr Institute/Usher Institute; University of Edinburgh Centre for Clinical Brain Sciences, Salvesen Mindroom Centre research centre for Learning Difficulties<br>Frank, John; University of Edinburgh, Farr Institute/Usher Institute
Keywords:	Child Abuse, Epidemiology, Child Psychology

SCHOLARONE™
Manuscripts

Factors associated with Adverse Childhood Experiences in Scottish children: a prospective cohort study

Louise Marryat[1], BA (hons.), MA, Ph.D and John Frank[1], B.Sc. (Hon), M.Sc, M.D. (Honours), C.C.F.P., F.R.C.P.

Affiliations: [1] Farr Institute/ Scottish Collaboration for Public Health Research and Policy, University of Edinburgh, UK

Address correspondence to: Louise Marryat, SCPHRP, University of Edinburgh, 20 West Richmond Street, Edinburgh, EH8 9DX, UK louise.marryat@ed.ac.uk , 0131 651 1688.

Short title: Prevalence of ACEs in Scottish children

Funding source: LM is supported by the Farr Institute @ Scotland, which is supported by a 10-funder consortium: Arthritis Research UK, the British Heart Foundation, Cancer Research UK, the Economic and Social Research Council, the Engineering and Physical Sciences Research Council, the Medical Research Council, the National Institute of Health Research, the National Institute for Social Care and Health Research (Welsh Assembly Government), the Chief Scientist Office (Scottish Government Health Directorates), (MRC Grant No: MR/K007017/1). LM sits within, and JF is supported by, the core grant to SCPHRP from the MRC, with half that support from the Scottish Chief Scientist Office (MR/K023209/1)

Conflicts of interest: The authors have no conflicts of interest to declare.

Financial Disclosure: The authors have indicated they have no financial relationships relevant to this article to disclose.

Abbreviations:

ACEs – Adverse Childhood Experiences
DWP – department for Work and Pensions
GUS – Growing Up in Scotland study
PAR – population attributable risk
SIMD – Scottish Index of Multiple Deprivation

Table of contents summary: This paper explores Adverse Childhood Experiences (ACEs) using prospective cohort data from Scotland. Using rich cohort data, we investigate which factors are associated with ACEs.

What is known about the subject

Adverse Childhood Experiences (ACEs) have been found to be commonly reported across adult populations. Limited evidence suggests that higher levels of ACEs are found among adults who had a younger mother, and who live in more deprived neighbourhoods. Lower levels have been seen in older populations, white or Asian populations and among graduates.

Confidential: For Review Only

What this study adds

ACEs were associated with being male, low income, younger mothers and urban areas in a current child cohort.

Contributors statement: Dr Marryat designed the study, cleaned and analysed the data and drafted the final report. Prof. Frank advised on study design and analyses. Dr Marryat and Prof. Frank both read and agreed the final manuscript and agree to be accountable for all aspects of the work.

Patient and Public Involvement statement:
This paper uses secondary data analysis of the first seven sweeps of the Growing Up in Scotland Study. The original study was designed in conjunction with the Scottish Government, academics, and third sector organisations, such as the Scottish Book Trust. The current analyses have had input from the Scottish Government ACEs team, have been presented and discussed at an NHS event, as well as being discussed with a variety of third sector organisations representing and working with people who have experienced ACEs. Further dissemination of the work is planned to practitioners working with people who have experienced ACEs, as well as to the general public through social media and national media.

Abstract

Background and Objectives

Adverse childhood experiences (ACEs) have been associated with a range of poorer health and social outcomes throughout the life course, however, to date they have primarily been conducted retrospectively in adulthood. This paper sets out to determine the prevalence of ACEs at age eight in a recent prospective birth cohort, and examine associations between risk factors in the first year and cumulative ACEs.

Design

This study uses the Growing Up in Scotland Birth Cohort 1, in which children born in Scotland in 2004/5 were identified using Child Benefit Records, and followed up for 7 years (n.3119). ACE scores and sample characteristics were calculated and described. Logistic regression models were fitted to explore associations between risk factors (sex, mother's age and education, household income, area level deprivation and urban/rural indicator) and ACE scores.

Results

Seven ACEs (or proxies) were assessed: physical abuse, domestic violence, substance abuse, mental illness, parental separation, parental incarceration, and emotional neglect. Instances of sexual abuse were too few to be reported. Emotional abuse and physical neglect could not be gathered. Around two-thirds of children had experienced one or more ACE, with 10% experiencing three or more in their lifetime. Higher ACE scores were associated with being male, having a young mother, low income, and urban areas.

Confidential: For Review Only

Conclusions

Using prospective data, the majority of children born in 2004/5 in Scotland experienced at least one ACE by age eight, although three ACEs could not be assessed in this cohort. ACEs were highly correlated with socio-economic disadvantage in the first year of life.

Confidential: For Review Only

INTRODUCTION

The Adverse Childhood Experiences (ACEs) scale was first explored with US adults, who were asked a series of questions covering childhood psychological, physical and sexual abuse, and household dysfunction. Around half (52.1%) reported experiencing at least one item[1]. Evidence from England and Wales, respectively, showed similar results [2 3]. A study from New Zealand exploring ACEs in the 1970's suggested that c.58% in a prospective study and c.65% in a retrospective study experienced at least one ACE [4].

Living in adverse socio-economic circumstances during childhood has a demonstrated association with later physical and mental health outcomes [5-7]. ACEs go beyond this to look at other adversities, e.g. abuse and parental imprisonment. Whilst there is likely to be a substantial overlap with deprivation, this is generally unknown, although evidence of associations with individual measures does exist [5 8 9]. The original ACE study included only adults with private health insurance, suggesting that, as adults, this group were relatively affluent [1]. Neighbourhood deprivation has been associated with increased levels of ACEs[10], however Bellis et al. only found deprivation to be associated with having four or more ACEs[11]. Higher levels of ACEs have been associated with having a younger mother[11], whilst lower levels have been found among older people, white or Asian people, and graduates[1].

ACEs have been linked to adverse outcomes in childhood and adulthood [1 2 12 13]: they have been associated with poorer self-rated health, premature mortality, suicide attempts, depression, ischemic heart disease, cancer, chronic lung disease, skeletal fractures, liver disease, fetal death, and Chronic Obstructive Pulmonary Disease[1 13-17]. ACEs have also been associated with risky lifestyle behaviours, e.g. drug and alcohol use, smoking, and high numbers of sexual partners [1], as well as job, financial, and reproductive problems[15]. They have been linked to multimorbidity in adulthood [1].

Confidential: For Review Only

Despite the current interest in ACEs and their seeming importance in relation to subsequent outcomes, we know little about modern ACEs. The capturing of ACEs in the general population is difficult, and relies on retrospective data collected in adulthood. The aims of this paper are to explore to what extent ACEs could be determined using prospective cohort data, and what prevalence levels look like in a recent population. This approach allows exploration of the predictors of ACEs, normally not available in retrospective data.

METHOD

Child and parent interview data were taken from the first seven waves of the Growing Up in Scotland (GUS) Study, covering the first eight years of the child's life. This study, funded by the Scottish Government, tracks the lives of children from birth through to their teenage years and beyond, and collects a wide range of information, including cognitive, social, emotional and behavioural development, health and wellbeing, childcare, education, and parenting and social networks. The full design has been reported elsewhere[18]. The sample was taken from child benefit records, which at the time of sampling included 97% of the Scottish population with children. Data zones (the key small-area geographic statistic in Scotland, each containing 500–1000 people and nested within Local Authorities) were aggregated until each area had an average of 57 live births per year, based on the previous three years of data, which was estimated to provide the required sample size. These Primary Sampling Units were then stratified by Local Authority (n.32) and then by Scottish Index of Multiple Deprivation Score (a measure of relative area level deprivation), before 130 points were chosen at random. Prior to final sampling, the Department of Work and Pensions removed any 'sensitive cases' (e.g. where there had been a death in the immediate family) and any cases which had been sampled in the previous 3 years (c.5%). They then sampled all babies within the selected points which met the date of birth criteria. In cases where more than one child met the criteria, one child was selected at random. Where children were found not to be living with their natural parent, they were followed up where possible in foster care or kinship care. Sweep 1 took place in 2005/6 when the children

https://mc.manuscriptcentral.com/bmjpo

were 10 months old with 5,217 children, comprising 81% of the eligible children[19]. At Sweep 7 3,456 children remained (66% of sweep 1 children), with a target interview date of 94.5 months old[18]. Table 1 details the missing cases. Cases were only included were the participant had the relevant ACE and demographic data, of which there were 3119 (90.2% of all Sweep 7 subjects). Exploratory analyses indicated that respondents missing data at this stage were more likely to be younger mothers, those living in more deprived areas, and those in urban areas. Cases without full data were not used: no imputation was used. Longitudinal weights, produced by the survey team at ScotCen were used, and the stratification of the sample was accounted for using the Complex Samples module within SPSS24[20]. Full information on weighting can be found in the data user guide[18.]

Confidential: For Review Only

154 **Table 1 Proportion of children who were included or missing at sweep 7 by**
155 **demographic characteristics**

	**Missing from sample (%)**	**Included in sample (%)**
Family type		
Lone parent	29.7	13.2
Living with partner	70.3	86.8
Age of mother at birth of cohort child		
Under 20	12.6	3.8
20-29	49.5	35.6
30-39	35.3	56.6
40 or older	2.6	4.0
Income quintile		
Bottom quintile	31.0	14.5
2	22.7	19.3
3	16.0	19.5
4	16.2	24.4
Top quintile	14.1	22.3
Scottish Index of Multiple Deprivation Quintiles		
5 – Most deprived	32.1	17.5
4	19.4	17.1
3	16.7	21.7
2	17.7	21.6
1 – Least deprived	14.2	22.1
Education level of mother		
No qualifications	14.7	6.2
Standard Grades or above	26.1	15.1
Highers or above	59.2	78.7
Child ethnic group		
White	93.5	96.5
Non-White	6.5	3.5
Child sex		
Male	53.1	50.6
Female	46.9	49.4
Urban/rural indicator		
Large urban	40.1	36.7
Other urban	34.6	29.4
Small accessible towns	9.2	9.8
Small remote towns	2.5	3.2
Accessible rural	10.5	15.4
Remote rural	3.1	5.4

156

Confidential: For Review Only

Base	*1761*	*3456*

Data from GUS were mapped onto the ACE questions (Supplementary Table 1) and socio-demographic information from the first sweep were combined into one dataset. Since ACE-related questionnaire items were asked inconsistently throughout the seven sweeps of the study, an 8-year cumulative incidence of ACEs was derived by summing the ACEs present for each child throughout the study period. Seven ACEs (or proxies for them), were available: physical abuse, domestic violence, substance abuse, mental illness, parental separation, parental incarceration, and emotional neglect. Information on Emotional abuse and Neglect was not available. Sexual abuse was reported in response to an open-ended question, however personal communications with the survey team revealed that fewer than five participants had responded positively to this, and it was thus excluded.

Ethics

Each sweep of data collection was subject to medical ethical review by the Scotland 'A' MREC committee. All parents interviewed (and Children, for the child interview) gave their informed consent prior to inclusion in the study. The current analyses were subject to ethical review in the University of Edinburgh.

Analyses

Binary variables of one or more ACE (vs. none) (1+ ACE) and three or more ACEs (vs. <3) (3+ ACEs) were derived. Three or more ACEs were explored, rather than four or more, due to the number of ACEs assessed being fewer and children having had less time to accumulate them.

Confidential: For Review Only

179 Socio-demographic data were described and Spearman correlations between them were explored.
180 All correlations were of a weak to medium strength (Supplementary Table 2).

181 Multiple univariable logistic regression models were fitted predicting the odds of having 1+ and
182 3+ACEs, respectively (Table 3). Predictors were taken from the current literature: sex, ethnicity,
183 income, age of mother at the birth of the first child and mother's educational qualifications.
184 Where univariable associations reached the criterion of $p<0.1$ these variables were put into the
185 multivariable model.

186 **Results**

187 Around two thirds (65%) of children experienced 1+ ACE, with 10% experiencing 3+ at age
188 eight (Figure 1).

189 *[FIGURE 1 ABOUT HERE]*

190 The most common ACEs were parental mental health problems (35.4%), and having parents
191 who were separated or divorced (32.1%). The proportion reporting frequent physical
192 punishment was high (22.2%). Few children experienced parental imprisonment (0.4%)
193 (Table 2).

194 **Table 2 Proportion of children within each ACE category**

ACE field	Weighted percentage
ACE1: emotional abuse	-
ACE2: corporal punishment	22.2%
ACE3: sexual abuse	-
ACE4: not loved or supported	20%
ACE5: neglect	-
ACE6: Parents ever divorced or separated	32.1%
ACE7: Domestic violence	9.1%

Confidential: For Review Only

ACE8: Drug or alcohol misuse	14.0%
ACE9: Mental health problems	35.4%
ACE10: Parent in prison	0.4%
Any ACE reported	65.0%
Base	*3119*

ACEs varied by household income quintiles: 1% of children in households in the top income quintile had 4+ACEs, compared with 10.8% in the lowest income group (Table 3).

Boys were more likely to experience 3+ ACEs, as were those with younger mothers, mothers with fewer educational qualifications, and children living in more deprived areas. A higher proportion of children from a White UK background, compared with other ethnic groups, experienced 3+ ACEs, although the low proportion (4.3%) of children from non-White-UK backgrounds limited the power of this comparison. Children in urban areas were more likely to have experienced a higher number of ACEs (3.4% vs 1.3% in rural areas).

Confidential: For Review Only

Table 3 Proportion of children in each ACE category by demographics

	Sample size	No. of ACES (%) None	1	2	3	4 or more	*Chi sq. p value*
Sex							
Male	*1609*	32.9	34.6	20.4	8.4	3.8	*0.004*
Female	*1510*	37.3	35.1	18.8	6.5	2.2	
Ethnicity							
White	*2982*	34.7	34.7	19.9	7.6	3.1	*0.07*
Non-white	*135*	43.6	36.7	13.4	5.6	0.6	
Education of mother							
Degree or higher	*842*	51.4	32.9	11.8	2.8	1.1	*<0.001*
Vocational qualification	*1163*	35	35.9	19.2	7.9	2	
Highers	*240*	36.4	32.2	24.9	5.9	0.6	
Standard Grade	*555*	18.7	38.2	23.3	12.3	7.4	
No qualifications	*291*	15.6	32.5	33.3	11.9	6.7	
Other	*24*	56.3	30.7	8	5	0	
Age of mother at birth of 1st child							
Under 20	*541*	12.5	30.1	31.5	16.3	9.5	*<0.001*
20 to 29	*1590*	34.8	35.9	20.1	7.0	2.3	
30 to 39	*945*	48.3	36.2	11.4	3.5	0.6	
40 or over	*35*	46.4	28.8	20.2	2.4	2.2	
Household equivalised income quintile							
Bottom (£<8,410)	*623*	8	27	34.5	19.7	10.8	*<0.001*
2nd quintile (£8,410-£13,749)	*587*	26.6	33.6	26.1	10.6	3.2	
3rd quintile (£13,750-£21,784)	*518*	33.8	38.6	18.3	8.3	1	
4th quintile (£21,785-£33,570)	*592*	42.2	36	17.1	4.2	0.5	
Top quintile (£>=£33,571)	*513*	52.8	32.7	11.1	2.3	1	
Scottish Index of Multiple deprivation (SIMD) area quintile							
1 – Most deprived	*745*	17.2	37.6	29.1	10.9	5.3	*<0.001*
2	*583*	30.3	35.9	19.2	10.9	3.7	
3	*637*	34.6	35.3	22	5.6	2.5	
4	*593*	43	35.2	14.6	5.3	2.0	
5 – Least Deprived	*561*	55.7	29.3	10.2	3.8	1.0	
Urban/rural classification							
Urban	*2567*	32.6	34.9	20.8	8.3	3.4	*<0.001*
Rural	*552*	46.3	34.5	14.3	3.6	1.3	

Independent associations were explored between risk factors in the first year of life and having 1+, or 3+ACEs, respectively. The multivariable model indicated that risk factors predicting having 1+ ACE at age eight were living in a lower income group, in particular living in the lowest income quintile (Odds Ratio 7.11);being male; having a mother with lower educational qualifications; having a mother who was under 20 or over 40 at the birth of

Confidential: For Review Only

her first child; living in an area with higher levels of deprivation; or in an urban area. (Table 4).

The model predicting having 3+ACEs at age 8 was very similar. In this multivariable model, maternal educational qualifications did not meet our criteria for statistical significance, although it is worth noting that the odds ratios were similar to the previous model. Once again, the strongest predictor of experiencing 3+ACEs was living in a household in the lowest income quintile, where odds were 5.7 times higher (Table 4).

Confidential: For Review Only

Table 4 Univariable and Multivariable logistic regression models predicting having 1+, and 3+, ACEs, respectively, at age 8.

	Univariable: 1+ ACE		*Multivariable 1+ ACE*		*Univariable: 3+ ACE*		*Multivariable 3+ ACE*	
	Odds ratio	*95% confidence interval*	*Odds ratio*	*95% confidence interval*	*Odds ratio*	*95% confidence interval*	*Odds ratio*	*95% confidence interval*
Sex of child								
Male	1.21	1.03-1.44	1.31	1.09-1.56	1.45	1.11-1.89	1.51	1.15-1.98
Female	-	-	-	-	-	-	-	-
Education of mother								
Degree or higher	0.18	0.11-0.27	0.87	0.54-1.41	0.18	0.10-0.33	0.86	0.38-1.92
Vocational qualification	0.34	0.22-0.53	1.07	0.67-1.73	0.48	0.30-0.79	1.10	0.58-2.10
Highers	0.32	0.20-0.53	0.94	0.55-1.63	0.30	0.13-0.67	0.43	0.16-1.16
Standard Grade	0.80	0.51-1.27	1.72	1.07-2.78	1.08	0.67-1.73	1.56	0.87-2.78
Other	0.14	0.05-0.43	0.41	0.12-1.45	0.23	0.03-1.85	0.54	0.05-6.53
No qualifications	-	-	-	-	-	-	-	-
Age of mother at birth of child								
Under 20	6.07	3.16-11.66	1.44	0.69-3.01	7.26	1.58-33.37	2.19	0.45-10.57
20 to 29	1.62	0.87-3.02	0.75	0.37-1.52	2.12	0.49-9.16	1.06	0.23-4.82
30 to 39	0.93	0.49-1.75	0.74	0.35-1.59	0.88	0.20-3.88	0.70	0.15-3.25
40 or over	-	-	-	-	-	-	-	-
Household equivalised income quintile								
Bottom (£<8,410)	15.05	9.67-23.43	7.11	4.53-11.17	11.99	7.02-20.47	5.73	2.82-11.64
2nd quintile (£8,410-£13,749)	3.16	2.36-4.22	1.96	1.52-2.53	3.90	2.12-7.16	2.45	1.15-5.20
3rd quintile (£13,750-£21,784)	2.07	1.59-2.69	1.52	1.17-1.97	2.97	1.70-5.20	2.26	1.17-4.35
4th quintile (£21,785-£33,570)	1.59	1.23-2.05	1.38	1.07-1.79	1.46	0.80-2.65	1.31	0.71-2.41
Top quintile (£>=£33,571)	-	-	-	-	-	-	-	-
Scottish Index of Multiple deprivation (SIMD) area quintile								
1 – Most deprived	6.06	4.71-7.80	2.29	1.71-3.06	1.56	0.89-2.72	1.05	0.60-1.84
2	2.90	2.25-3.73	1.47	1.07-2.01	1.75	1.00-3.05	1.19	0.66-2.16
3	2.38	1.93-2.94	1.83	1.42-2.36	3.51	2.02-5.75	0.98	0.54-1.80

Confidential: For Review Only

4	1.67	1.31-2.13	1.52	1.15-2.02	3.84	2.33-6.34	1.22	0.73-2.04
5 – Least Deprived	-	-	-	-	-	-	-	-
Urban/rural classification								
Urban	1.79	1.50-2.13	1.57	1.26-1.95	2.56	1.62-4.05	1.82	1.14-2.92
Rural	-	-	-	-	-	-	-	-
Ethnicity								
White	1.46	0.95-2.48	-	-	1.80	0.80-4.03	-	-
Non-white	-	-	-	-	-	-	-	-
Sample size	*2848*		*2848*		*2848*		*2848*	

Confidential: For Review Only

Discussion

By eight years, around two-thirds of children had 1+ ACE, with one in ten children experiencing 3+. Results indicate that children living in more disadvantaged circumstances were more likely to experience ACEs than their more privileged peers.

Strengths

This paper is unique in using a range of prospective data on ACEs in a current generation of children. GUS covers a representative sample of children from Scotland, providing large enough numbers to explore ACEs by subgroups, combining a mixture of parental and child-reported data, giving a rounded picture of ACEs which have occurred. The inclusion of family background data allows us insight into what early predictors are associated with having higher odds of experiencing ACEs. In addition, families were asked to answer a range of questions about their lives, rather than a specific questionnaire about ACEs: Felitti et al. found that respondents to the ACE questionnaire were slightly more likely to have reported sexual abuse in a separate questionnaire, suggesting that there may be a bias within the original ACE studies towards people who had experienced ACEs[1].

Weaknesses

The original ACE questionnaire has not yet been included in GUS and so some proxy information had to be used, which may not accurately reflect the ACE questionnaire. Two ACEs (emotional abuse and neglect) were not able to be assessed through the GUS questions. In addition, the proportion of parents reporting sexual abuse was so low that we were not able to include this. It is possible that social desirability may lead to some parents not disclosing where ACEs had occurred and, furthermore, if ACEs, such as abuse, happened outside the home, parents may not be aware that they have occurred. All these factors are likely to lead to this study underestimating the prevalence of ACEs. Despite the sample being representative of the population of children in Scotland, some particularly sensitive cases were removed

Confidential: For Review Only

Figure 1 Number of ACEs reported at age eight

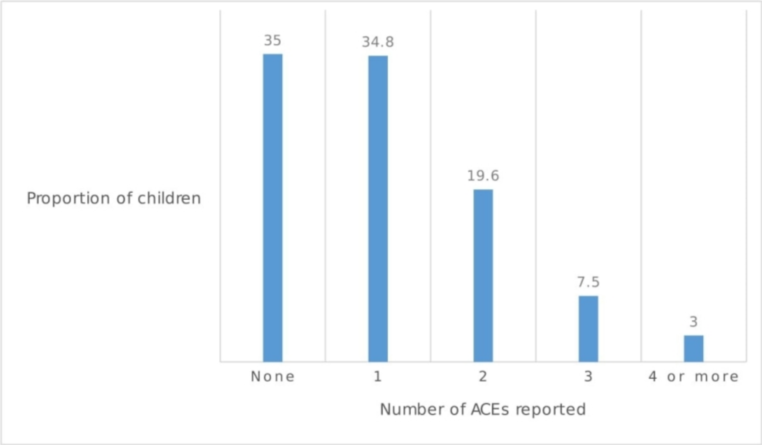

Sample size: 3119

133x131mm (300 x 300 DPI)
